# Human Antibodies that Slow Erythrocyte Invasion Potentiate Malaria-Neutralizing Antibodies

**DOI:** 10.1016/j.cell.2019.05.025

**Published:** 2019-06-27

**Authors:** Daniel G.W. Alanine, Doris Quinkert, Rasika Kumarasingha, Shahid Mehmood, Francesca R. Donnellan, Nana K. Minkah, Bernadeta Dadonaite, Ababacar Diouf, Francis Galaway, Sarah E. Silk, Abhishek Jamwal, Jennifer M. Marshall, Kazutoyo Miura, Lander Foquet, Sean C. Elias, Geneviève M. Labbé, Alexander D. Douglas, Jing Jin, Ruth O. Payne, Joseph J. Illingworth, David J. Pattinson, David Pulido, Barnabas G. Williams, Willem A. de Jongh, Gavin J. Wright, Stefan H.I. Kappe, Carol V. Robinson, Carole A. Long, Brendan S. Crabb, Paul R. Gilson, Matthew K. Higgins, Simon J. Draper

**Affiliations:** 1 The Jenner Institute, University of Oxford, Old Road Campus Research Building, Oxford OX3 7DQ, UK; 2 Department of Biochemistry, University of Oxford, South Parks Road, Oxford OX1 3QU, UK; 3 Burnet Institute, 85 Commercial Road, Melbourne, VIC 3004, Australia; 4 Department of Chemistry, University of Oxford, Oxford OX1 3QZ, UK; 5 Center for Global Infectious Disease Research, Seattle Children’s Research Institute, 307 Westlake Ave. N., #500, Seattle, WA 98109, USA; 6 Laboratory of Malaria and Vector Research, NIAID/NIH, Rockville, MD 20852, USA; 7 Cell Surface Signalling Laboratory, Wellcome Trust Sanger Institute, Cambridge CB10 1SA, UK; 8 ExpreS^2^ion Biotechnologies, SCION-DTU Science Park, Agern Allé 1, Hørsholm 2970, Denmark

**Keywords:** malaria, blood-stage, merozoite, structural vaccinology, RH5, synergy, monoclonal antibody, neutralization, X-ray crystallography, live-cell microscopy

## Abstract

The *Plasmodium falciparum* reticulocyte-binding protein homolog 5 (PfRH5) is the leading target for next-generation vaccines against the disease-causing blood-stage of malaria. However, little is known about how human antibodies confer functional immunity against this antigen. We isolated a panel of human monoclonal antibodies (mAbs) against PfRH5 from peripheral blood B cells from vaccinees in the first clinical trial of a PfRH5-based vaccine. We identified a subset of mAbs with neutralizing activity that bind to three distinct sites and another subset of mAbs that are non-functional, or even antagonistic to neutralizing antibodies. We also identify the epitope of a novel group of non-neutralizing antibodies that significantly reduce the speed of red blood cell invasion by the merozoite, thereby potentiating the effect of all neutralizing PfRH5 antibodies as well as synergizing with antibodies targeting other malaria invasion proteins. Our results provide a roadmap for structure-guided vaccine development to maximize antibody efficacy against blood-stage malaria.

## Highlights

• Human PfRH5 vaccination induces cross-reactive neutralizing antimalarial antibodies
• Neutralizing human PfRH5 antibodies bind epitopes close to the basigin binding site
• Some non-neutralizing antibodies potentiate those binding several malaria proteins
• Potentiating antibodies slow erythrocyte invasion by binding a new epitope on PfRH5

## Graphical Abstract

**Figure:**
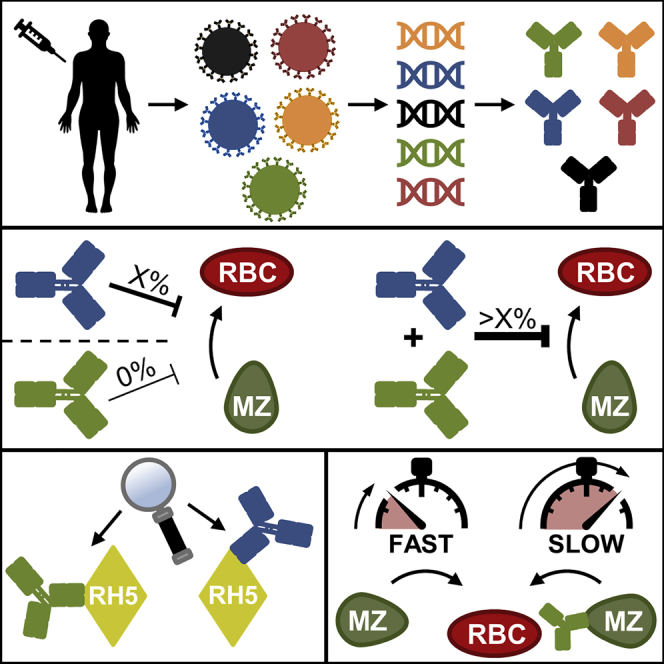

## Teaser

Analyses of human monoclonal antibodies against the *Plasmodium falciparum* protein PfRH5 identify a subset of non-neutralizing antibodies that synergize with a repertoire of other neutralizing antibodies by slowing the ability of malaria-causing parasites to invade red blood cells.

## Introduction

Malaria, responsible for some 435,000 deaths annually, is the biggest parasitic killer in the world today with *Plasmodium falciparum* accountable for the vast majority of these deaths (World Health Organization, 2018). Existing drugs and insecticides are effective control measures but require sustained and expensive investment to deploy and are threatened by the emergence of resistance. It is therefore widely accepted that an efficacious antimalarial vaccine, engendering adaptable and durable immunity, will be a key factor in driving this disease toward elimination and ultimate eradication. However, this has proved challenging, and efforts to generate vaccines that target the invasive merozoite in the disease-causing blood-stage of malaria infection have, to date, not been successful (Draper et al., 2018). Previously, the advancement of leading blood-stage subunit vaccine candidates has been impeded by redundant invasion pathways (Wright and Rayner, 2014), considerable sequence polymorphism in target antigens (Takala et al., 2009), and the elicitation of antibody responses in human vaccinees of insufficient magnitude and/or breadth for effective neutralization (Draper et al., 2018). This has raised the imperative to identify new conserved and essential vaccine immunogens, to discover the most effective epitopes of these immunogens for protective human antibodies and to design molecules that will elicit these antibodies to produce the most effective immune response.

Central to the symptomatic blood-stage of malaria infection is the cyclical infection of host red blood cells (RBC) by the merozoite form of the parasite. A fundamental and non-redundant event in this process is the binding of *P. falciparum* reticulocyte-binding protein homolog 5 (PfRH5) on the merozoite to its host RBC receptor basigin (BSG) (Crosnier et al., 2011). Although the precise function of PfRH5 is not known, it is linked to calcium influx into the erythrocyte, followed by cytoskeleton remodeling and is necessary for establishing a tight junction between parasites and RBCs (Weiss et al., 2015, Volz et al., 2016). Invasion is accompanied by an N-terminal processing event of unknown function, which trims PfRH5 from ∼60 kDa to ∼45 kDa (Baum et al., 2009). PfRH5 associates with other merozoite surface proteins to form an essential (Volz et al., 2016) invasion complex including cysteine-rich protective antigen (PfCyRPA) (Reddy et al., 2015), PfRH5-interacting protein (PfRipr) (Chen et al., 2011), and glycosylphosphatidylinositol (GPI)-linked PfP113 (Galaway et al., 2017).

Several additional attributes of PfRH5 make it an attractive vaccine candidate. Despite its uncommon protein fold (Wright et al., 2014, Chen et al., 2014), PfRH5 can be expressed as a soluble recombinant protein in several systems including mammalian HEK293 cells (Crosnier et al., 2011), insect cells (Chen et al., 2014, Hjerrild et al., 2016), and *E. coli* following protein engineering (Campeotto et al., 2017). Furthermore, low levels of antibodies elicited by repeated natural infection (Douglas et al., 2011) suggest that neutralizing antibodies that target PfRH5 in naturally acquired responses are rare. Low natural immune pressure, coupled with functional constraints linked to BSG binding (Wanaguru et al., 2013), likely account for the limited sequence diversity of PfRH5 (Manske et al., 2012). Blood-stage malaria vaccinology benefits from the use of an established *in vitro* assay of growth inhibition activity (GIA) (Miura et al., 2009) that correlates with vaccine-induced (as opposed to naturally acquired) protection in non-human primate (NHP) malaria infection models (Singh et al., 2006, Mahdi Abdel Hamid et al., 2011, Douglas et al., 2015) and successfully predicts protection against *P. falciparum* in a humanized mouse model and *Aotus* monkeys (Foquet et al., 2018, Douglas et al., 2019). In this assay, PfRH5-specific immunoglobulin G (IgG) antibodies are effective against all *P. falciparum* parasite strains and isolates tested in preclinical settings (Douglas et al., 2011, Williams et al., 2012, Bustamante et al., 2013, Reddy et al., 2014). Nevertheless, a combination of *in vitro* growth inhibition studies and the *Aotus* monkey *in vivo* challenge trials suggest that high concentrations of PfRH5-specific polyclonal IgG (>300 μg/mL) are likely needed for effective immunity. Such antibody levels will be challenging to achieve and sustain by human-compatible vaccination regimens (Payne et al., 2017) and to date, no human trial of a blood-stage malaria vaccine has achieved the predicted threshold of clinical efficacy (>60% GIA at a 1:4 serum dilution) (Singh et al., 2006, Douglas et al., 2015). However, in recent years, rational structure-informed vaccine design strategies have been developed to address this challenge. These approaches immuno-focus vaccine-induced responses, specifically seeking to elicit the most protective antibodies (McLellan et al., 2011, Correia et al., 2014, Pierce et al., 2017). Understanding the key protective epitopes on PfRH5 recognized by human IgG is likely to allow similar approaches to be used to reduce the required specific polyclonal antibody (pAb) concentration to tractable levels.

Here, we examine a panel of human monoclonal antibodies (mAbs) to PfRH5, isolated from the first clinical trial of a PfRH5-based vaccine (Payne et al., 2017), to define critical mAb epitopes in atomic detail. Furthermore, we explore the functional interplay between classes of mAbs likely to be contained in pAb targeting PfRH5 following vaccination and highlight the implications of this for rationally designed next-generation blood-stage malaria subunit vaccines.

## Results

### Vaccine-Induced Human mAbs to PfRH5

**Figure S1:**
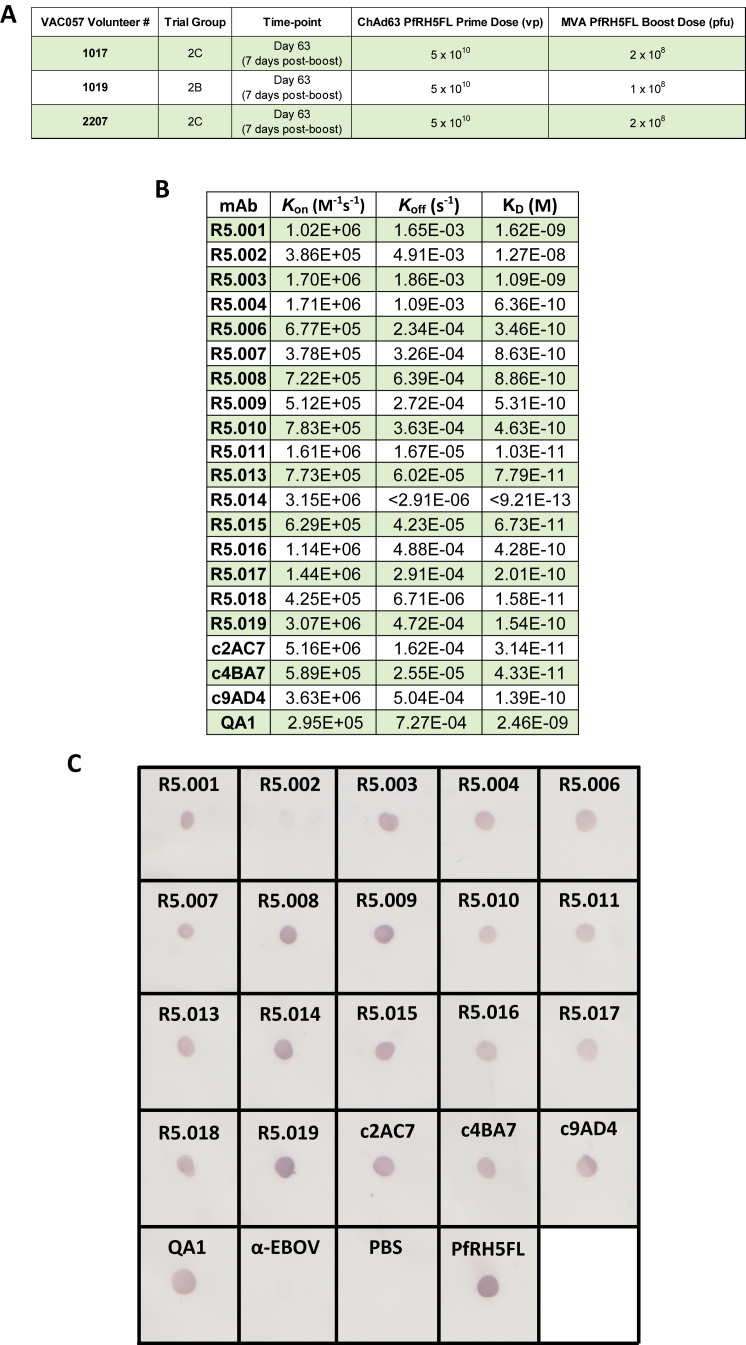
Clinical Trial (VAC057) Volunteer Information and Additional PfRH5 mAb Binding Data, Related to Figure 1 (A) Vaccination details of the three volunteers from which anti-PfRH5FL mAbs were isolated. (B) List of anti-PfRH5 mAb binding properties. Association-rate (*K*_on_), dissociation-rate (*K*_off_) and affinity (K_D_) are shown. (C) Dot blot showing anti-PfRH5 mAbs binding to parasite-derived PfRH5. PBS, PfRH5FL and a recombinant anti-*Ebolavirus* IgG1 mAb (α-EBOV) were used as controls.

**Figure 1:**
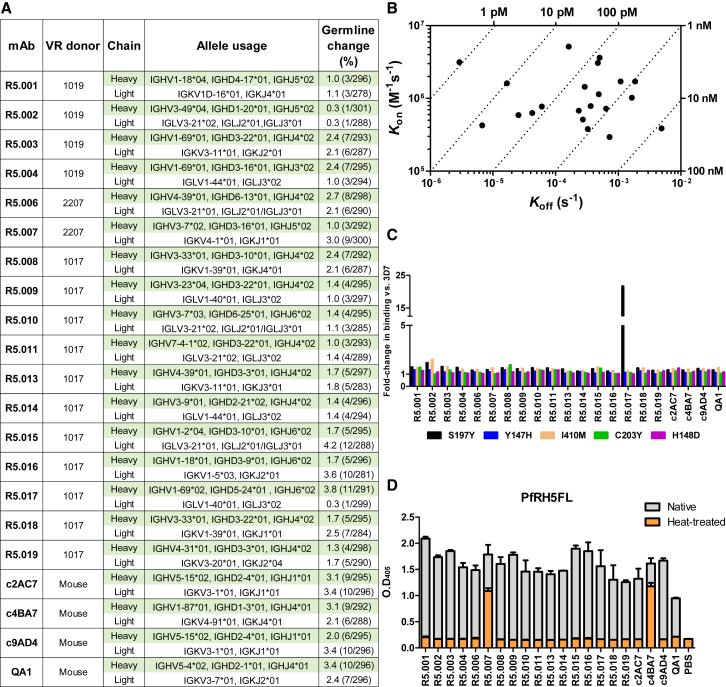
Description and Binding Characteristics of Anti-PfRH5 mAbs (A) Genetic lineage of variable regions from PfRH5-specific mAbs and their donor origin, showing percentage of nucleotide substitutions relative to germline. (B) Iso-affinity plot showing kinetic rate constants for binding of mAbs to PfRH5FL as determined by SPR. Diagonal dotted lines represent equal affinity at equilibrium. (C) Real-time analysis by BLI of mAb binding to PfRH5FL variants with the five most common naturally occurring amino acid substitutions. Bars represent fold-change compared to wild type PfRH5FL (3D7 sequence) binding. (D) Assessment of binding of PfRH5-specific mAbs to heat-treated PfRH5FL protein by ELISA. Bars show the mean and error bars show the SEM (n = 2). See also Figure S1.

Anti-PfRH5 mAbs were isolated from single-cell-sorted plasmablasts of immunized volunteers enrolled in a first-in-human Phase Ia clinical trial of a PfRH5-based vaccine delivered using recombinant chimpanzee adenovirus and poxvirus viral-vectors (Figure S1A) (Payne et al., 2017). Variable region (VR)-coding genes were isolated by RT-PCR and PCR and cloned into a human IgG1 scaffold. Cognate heavy-chain and light-chain plasmids were co-transfected in HEK293 cells, and PfRH5-specificity was confirmed by supernatant reactivity to full-length PfRH5 protein comprising amino acids E26–Q526 (PfRH5FL) by ELISA. Seventeen genetically distinct mAbs were isolated (Figure 1A). Alignment with the most similar germline VR gene segment alleles in IgBLAST (Ye et al., 2013) revealed little non-germline sequence, suggesting that PfRH5 is readily recognized with high affinity by germline B cell receptors in humans. The monovalent binding affinity of each mAb was assessed by surface plasmon resonance (SPR), with anti-PfRH5 mouse or chimeric (c) mAbs c2AC7, c4BA7, c9AD4, and QA1 included because of their extensive previous characterization (Douglas et al., 2014, Wright et al., 2014) (Figures 1B and S1B). Affinities were in the low nanomolar to high picomolar range.

The effect of PfRH5 polymorphism on mAb recognition was determined by measuring binding to recombinant PfRH5FL variants, each carrying one of the five most common naturally occurring amino acid substitutions (Figure 1C). In each case, except for C203Y, the generated PfRH5FL variants carried the minor allele. All global minor allele frequencies were below 0.19 (MalariaGEN v4.0) (Manske et al., 2012). The only mAb to show significant differential binding was R5.017, for which binding was reduced by S197Y. Only one human- and one mouse-derived mAb (R5.007 and c4BA7) were able to bind heat-treated PfRH5FL protein, suggesting a linear epitope (Figure 1D). In addition, a dot blot against *P. falciparum* 3D7 clone *in vitro* culture supernatant showed all mAbs to bind parasite-expressed PfRH5 (Figure S1C) that consists of a processed ∼45 kDa form (Baum et al., 2009, Chen et al., 2011).

### The Neutralizing Capacity of Human mAbs Binding PfRH5

**Figure 2:**
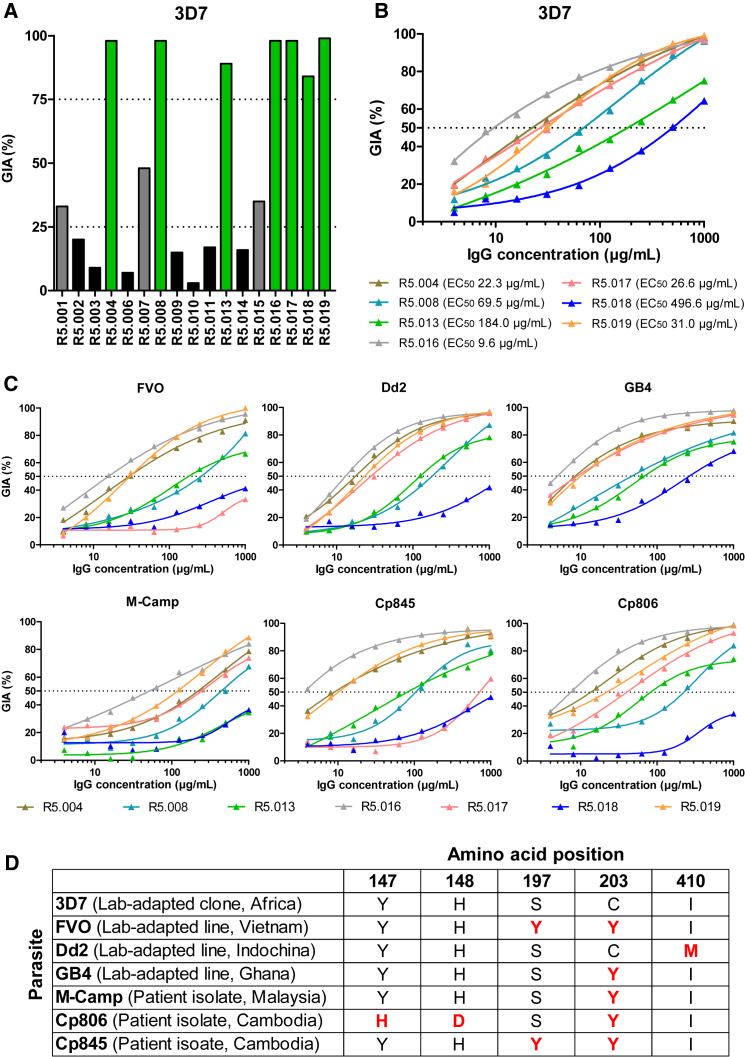
Growth Inhibitory Properties of Human PfRH5-Specific mAbs (A) *In vitro* GIA of each mAb tested at 3 mg/mL against 3D7 clone *P. falciparum*. Bars are color-coded to reflect potency (“GIA-high” [≥75%] in green, “GIA-low” [75% > GIA > 25%] in gray and “GIA-negative” [≤25%] in black). (B) *In vitro* GIA dilution series against the 3D7 reference clone. EC_50_ values were determined by interpolation after fitting data to a four-parameter dose-response curve. (C) *In vitro* GIA dilution series of GIA-high mAbs against heterologous parasite laboratory lines and isolates. (D) Amino acids at the five most common polymorphic sites of PfRH5 for the seven parasite lines. Deviations from the 3D7 reference sequence are highlighted in red. All GIA data points are the mean of duplicate wells, with each dataset fitted to a four-parameter dose-response curve. See also Figure S2.

**Figure S2:**
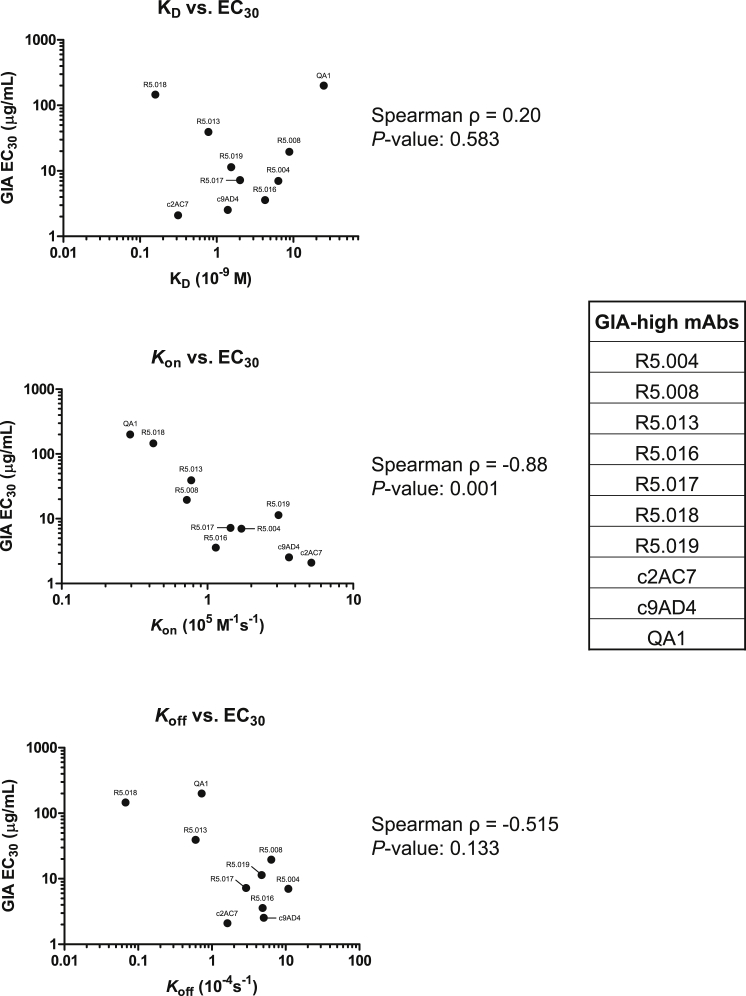
Correlation between nAb Kinetic Binding Parameters and GIA, Related to Figures 2 and S1B The binding parameters of nAbs (*K*_on_, *K*_off_ and K_D_) were correlated with growth inhibition using the GIA EC_30_ as a measure of potency. Only GIA-high nAbs were included in this analysis because of their overt ability to bind merozoite-bound PfRH5, as evidenced by their growth inhibitory properties. EC_30_ values were calculated from data shown in Figure 2B. Reported *P*-values are two-tailed and considered significant if *P* <0.05.

To characterize the ability of the human mAbs to block merozoite entry into RBCs, they were tested for *in vitro* GIA against 3D7 clone *P. falciparum*. An initial screen was carried out at high concentration, grouping the mAbs into three categories: “GIA-high” (GIA ≥75%), “GIA-low” (75% > GIA > 25%), and “GIA-negative” (GIA ≤25%) (Figure 2A). Dilution curves of GIA-high mAbs were made against 3D7 clone parasites to assess potency (Figure 2B). The two most potent mAbs (R5.016 and R5.004) had EC_50_ values comparable to the most potent anti-merozoite mouse-derived mAbs previously described (Douglas et al., 2014, Ord et al., 2014). A strong correlation was also observed between the association-rate (K_on_) of the neutralizing antibodies (nAb) and GIA (Figure S2), indicating a limited time window for mAb-PfRH5 binding in the context of merozoite invasion (Douglas et al., 2014, Saul, 1987). GIA assays were repeated against six heterologous strains and isolates originating from diverse geographical locations (Figure 2C), which contain all five of the most common PfRH5 polymorphisms (Figure 2D). This revealed some strain-dependent differences in anti-PfRH5 mAb potency, albeit with very similar hierarchies. Indeed, GB4 was more easily neutralized by all mAbs relative to 3D7, while M-Camp was less easily neutralized. The reason for these *in vitro* differences, and their relevance to *in vivo* neutralization, remains uncertain, as the M-Camp isolate was as susceptible as 3D7 to PfRH5 vaccine-induced polyclonal human IgG from the same origin as the mAbs (Payne et al., 2017). One notable exception was R5.017, which lacked efficacy against the FVO strain and Cp845 isolate. Only these two parasites carry the S197Y polymorphism (Figure 2D) that reduces binding of R5.017 (Figure 1C).

### Clustering of PfRH5-Specific mAbs into Functional Groups

**Figure S3:**
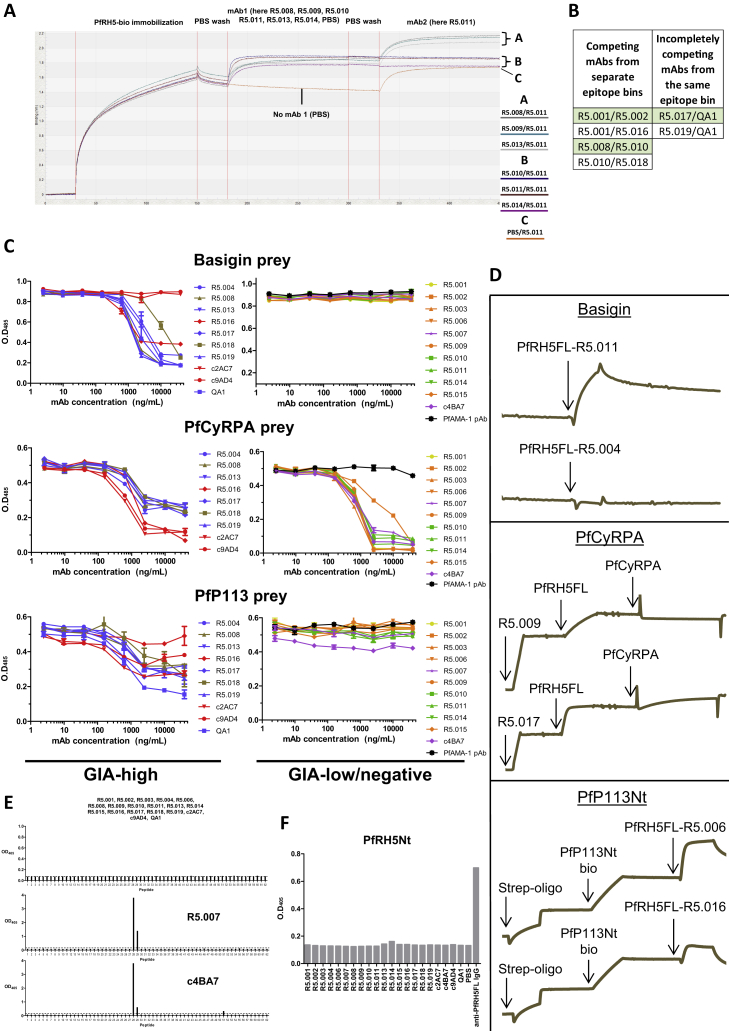
Further Investigations into Anti-PfRH5 mAb Binding Activity, Related to Figure 3 and Table S1 (A) Example graph of raw BLI data used to generate the epitope bins shown in Figure 3A. (B) Table of epitope bin exceptions. “Competing mAbs from separate epitope bins” are mAb pairs in which mAb1 was able to reduce mAb2 binding by ≥ 95% on a one-to-one basis but whose binding profile correlation coefficient was too low to reach the 0.7 threshold. “Incompletely competing mAbs form the same epitope bin” are mAb pairs in which mAb1 was not able to reduce mAb2 binding by ≥ 95% on a one-to-one basis but whose binding profile correlation coefficient was above the 0.7 threshold. (C) AVEXIS assays to determine mAb inhibition of PfRH5FL binding to associated proteins basigin, PfCyRPA and PfP113. In these plate-based assays, monobiotinylated PfRH5FL bait was coated to wells of streptavidin coated microtiter plates. Binding in the presence of each mAb was probed with pentameric prey proteins of either basigin, PfCyRPA or PfP113. Data points are the mean, error bars show the SEM. (D) Example graphs of raw SPR data used to make graphs in Figure 3C. Each assay setup was different for technical reasons. (E) Biotinylated 20-mer peptides overlapping by ten residues and spanning the whole PfRH5 sequence were assayed for mAb binding by ELISA on streptavidin-coated plates. Only two mAbs (R5.007 and c4BA7) were able to bind any of these overlapping peptides. (F) Binding of anti-PfRH5 mAbs to PfRH5Nt by ELISA. “Anti-PfRH5FL IgG” is PfRH5-vaccinated polyclonal IgG of human origin included as a positive control. Data in panels E and F are representative of singlicate wells.

**Figure 3:**
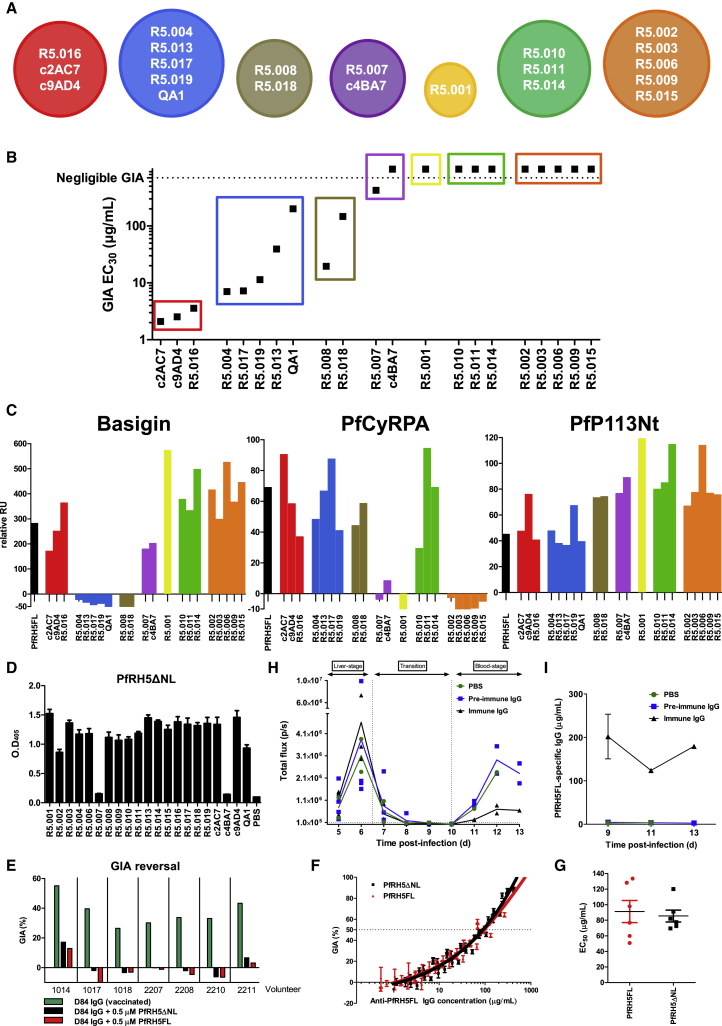
Epitope Binning Reveals that All Relevant Neutralizing Epitopes Lie within PfRH5ΔNL (A) Epitope bins determined by BLI from a matrix of sequential PfRH5FL-binding assays for different mAbs, with data used to construct these bins in Figure S3A and Table S1. (B) Potency of anti-PfRH5 mAb invasion inhibition grouped by epitope bin. EC_30_ values were interpolated from data in Figure 2B. (C) The effect of mAbs on binding of PfRH5FL to BSG, PfCyRPA, and PfP113Nt, as determined by SPR. Black bars show binding in the absence of mAb as a control. The colors used in (B) and (C) match those of the epitope bins in (A). (D) Binding of anti-PfRH5 mAbs to PfRH5ΔNL by ELISA. Bars show the mean of 4 replicate wells. (E) GIA of purified total IgG from seven different PfRH5FL-vaccinated human volunteers alone or with 0.5 μM of PfRH5FL or PfRH5ΔNL protein (30 μg/mL and 20 μg/mL, respectively). Bars show the mean of duplicate wells. (F) GIA of purified total IgG from rabbits immunized with PfRH5FL or PfRH5ΔNL (n = 6 rabbits per group). Concentrations of PfRH5FL-specific polyclonal IgG were measured by ELISA using a conversion factor determined by calibration-free concentration analysis. Individual data points show the mean of triplicate wells. (G) Comparison of EC_50_ for rabbit sera. EC_50_ values were determined for each rabbit by interpolation after fitting the data from (F) to a four-parameter dose-response curve. Black horizontal bars represent the mean. (H) Intravital luminescence signal of humanized mice infected with transgenic *P. falciparum* (NF54-luciferase) following passive transfer of 15 mg of pre-vaccination or PfRH5ΔNL-vaccinated rabbit IgG at day (d)6 post-infection. Starting groups were n = 2 for the PBS group and n = 4 for the IgG passive transfer groups. Mice that died before the experiment endpoint (d13) were: one at d7 and one at d13 in the PBS group, one at d9 and one at d12 in the pre-vaccination IgG group and one at d6, one at d10 and one at d12 in the vaccinated IgG group. Individual data points are connected by a line representing the mean. (I) Concentration time course of PfRH5-specific rabbit IgG in the passive transfer experiment shown in (H), as determined by ELISA binding to PfRH5FL. Data points show the mean. All GIAs used 3D7 clone *P. falciparum*. All error bars show the SEM.

To better understand the relationship between mAb binding site and function, mAbs were tested in pairs for simultaneous binding to monobiotinylated PfRH5FL by Bio-Layer Interferometry (BLI) (Figures S3A and S3B; Table S1). This defined seven distinct epitope bins, each containing mAbs that bind overlapping epitopes (Figure 3A). The resulting bins strongly correlated with GIA, with the red, blue, and olive bins containing the most potent anti-PfRH5 mAbs and the purple, yellow, green, and orange bins exclusively containing GIA-low or GIA-negative antibodies (Figure 3B). The epitope bins also proved successful in clustering together mAbs whose binding blocked *in vitro* interactions between PfRH5FL and the invasion complex protein PfCyRPA or the PfRH5 receptor BSG (Figures 3C, S3C, and S3D). Indeed, the blue and olive bins contained all mAbs that block BSG binding by both SPR and AVEXIS, while the purple, yellow, and orange bins contained all the mAbs that block PfCyRPA binding in the SPR assay and the most potent blockers in the AVEXIS assay. The lack of PfP113 blockade (Figure 3D) was unsurprising because no mAbs bound the PfRH5 N-terminal region (PfRH5Nt), which includes amino acids K33–K51 reported as the minimal PfP113 binding region (Galaway et al., 2017) (Figures S3E and S3F). Collectively, these data highlight the vicinity of the BSG binding site, and not the PfCyRPA binding site, as the key target of growth inhibitory mAbs, and show that the most potent nAbs bind to these regions with the highest association-rates (Figure S2).

### All Major Neutralizing PfRH5 Epitopes Are within the Truncated PfRH5ΔNL Protein

Previous crystallization of PfRH5 was aided by removal of two sections of disordered sequence resulting in a truncation lacking 188 of 526 residues (M1-Y139 and N248-M296), termed PfRH5ΔNL (Wright et al., 2014). Notably this truncated protein lacks PfRH5Nt (including the minimal PfP113 binding region) and thus a potential site for antibody-mediated neutralization. Interestingly, all GIA-high mAbs and two of three GIA-low mAbs bound PfRH5ΔNL, suggesting that this construct contains the major neutralizing epitopes (Figure 3D). Only R5.007 and c4BA7 were unable to bind this construct. Their binding site was mapped by ELISA to the internal disordered loop and not the N-terminal region, using an array of overlapping peptides. Both mAbs bound the same linear PfRH5 peptides (amino acids Y242–D261) (Figure S3E), consistent with their recognition of heat-treated PfRH5FL protein (Figure 1D). Furthermore, no mAbs bound recombinant PfRH5Nt by ELISA (amino acids F25–K140) (Figure S3F).

We next assessed the effect of PfRH5FL or PfRH5ΔNL proteins on the GIA of total polyclonal IgG purified from sera of PfRH5FL-immunized human vaccinees (Payne et al., 2017). The *in vitro* GIA could be reversed by adding 0.5 μM of recombinant PfRH5FL or PfRH5ΔNL into the IgG sample. GIA of IgG from all seven vaccinated volunteers was completely reversed following the addition of either PfRH5 variant protein (Figure 3E), a finding that also repeated when using purified IgG from rabbits immunized with PfRH5FL (data not shown). Notably, vaccine-induced IgG against PfRH5Nt was previously reported in the serum of these volunteers by ELISA (Payne et al., 2017), suggesting minimal or no contribution of these responses to overall GIA. To confirm that PfRH5ΔNL vaccination can also elicit antibody to the most functionally relevant neutralizing epitopes present in PfRH5FL, two groups of rabbits were immunized with equimolar doses of PfRH5FL or PfRH5ΔNL. The resulting purified IgG showed comparable potency in the GIA assay across both groups, indicating that no major neutralizing epitopes are lost in PfRH5ΔNL (Figures 3F and 3G). Passive transfer of PfRH5ΔNL-immunized rabbit IgG into humanized mice, carrying human hepatocytes and erythrocytes and challenged by *P. falciparum*-infected mosquito bites (Foquet et al., 2018), resulted in a significant reduction of blood-stage parasite burden, further serving to highlight that vaccine-induced IgG to this PfRH5 truncation is sufficient to effectively retard or arrest blood-stage parasitemia *in vivo* (Figure 3H). Analysis of PfRH5-specific pAb concentrations in the serum of passively immunized mice (Figure 3I) were consistent with the levels that led to control of blood-stage parasitemia, but not sterilizing immunity, in PfRH5FL-vaccinated *Aotus* monkeys (Douglas et al., 2015). The rigid α-helical core of PfRH5, lacking PfRH5Nt, is thus likely to contain all major neutralizing epitopes and is sufficient to replicate the neutralizing effect of antibody raised to the full-length PfRH5 antigen.

### Identifying the Epitopes for Key Neutralizing mAbs

**Figure 4:**
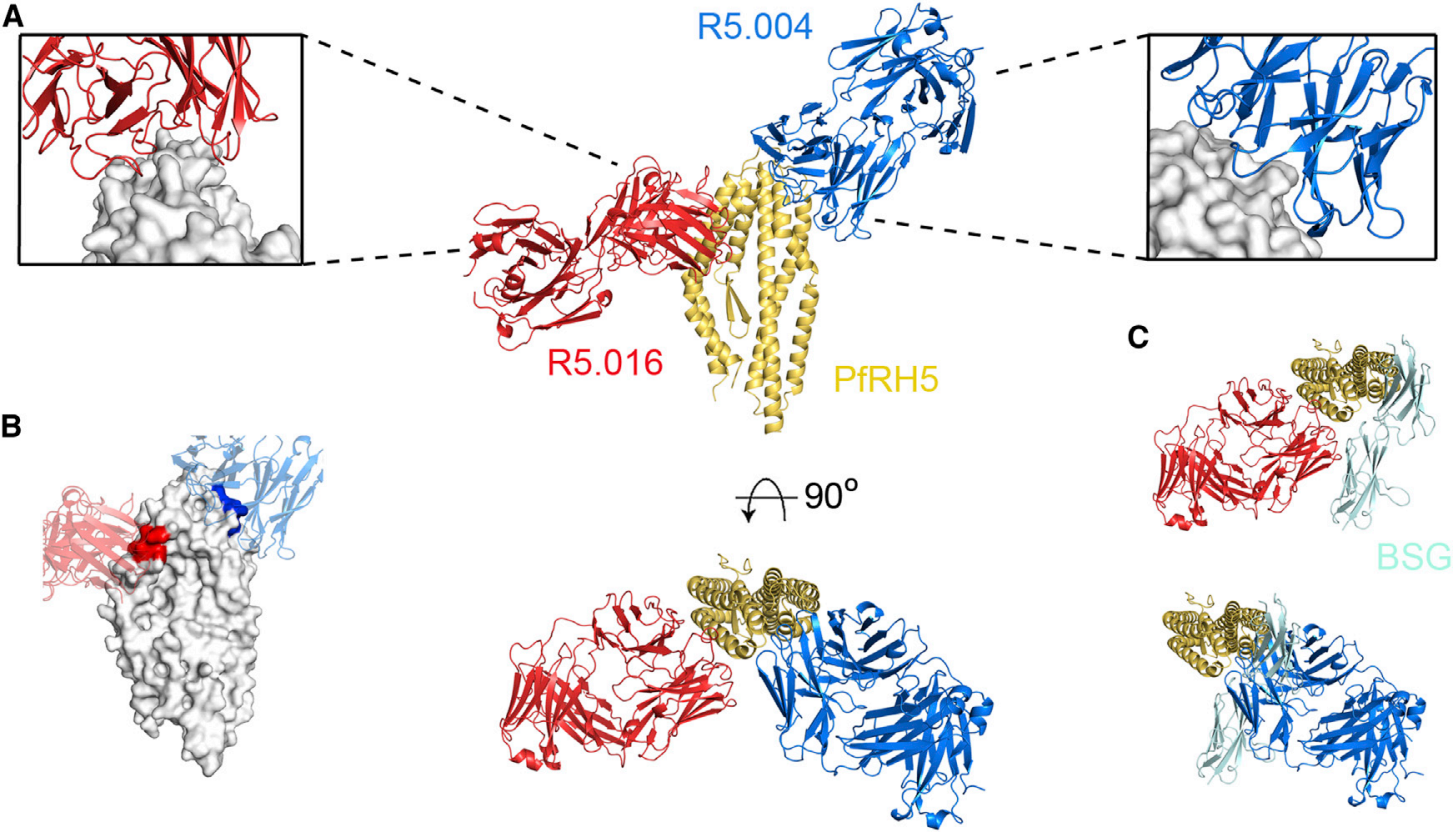
Structures of R5.004 and R5.016 Epitopes (A) Structure of PfRH5ΔNL bound to R5.004 and R5.016 Fab fragments. Insets show close-up views of epitopes. (B) The top PfRH5 peptides protected in HDX-MS by R5.004 mAb (blue) and R5.016 mAb (red) binding, are highlighted on the structure of PfRH5ΔNL. Positions of Fab fragments are overlaid as faded cartoons. (C) Overlay of PfRH5ΔNL:BSG (PDB: 4U0Q, BSG in teal) with R5.004 Fab or R5.016 Fab structures at their respective binding sites in the PfRH5ΔNL:R5.004:R5.016 structure. See also Figure S4 and Tables S2 and S3.

**Figure S4:**
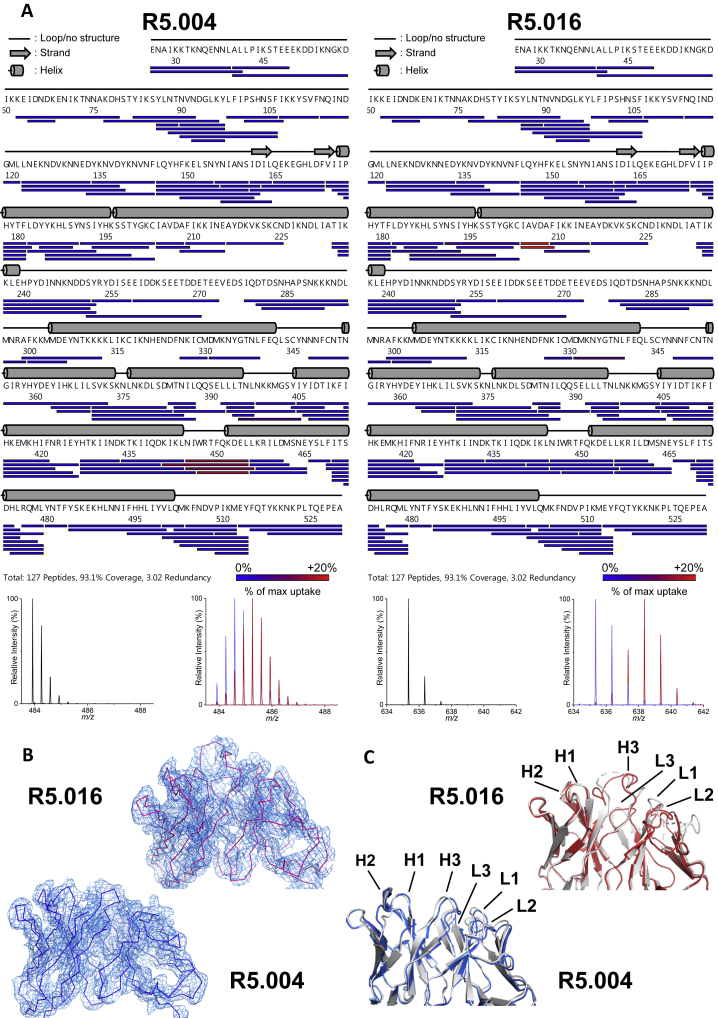
PfRH5 Peptide Protection in HDX-MS by R5.004 and R5.016 and Description of Bound R5.004 and R5.016 Fab Fragments, Related to Figure 4 (A) PfRH5 peptide map showing PfRH5FL protection from deuteration upon R5.004 (left) and R5.016 (right) binding. Secondary structure is shown in gray. Examples of highly protected peptides are shown in the mass spectra below (NIWRTFQKDEL for R5.004 and IAVDAF for R5.016). Black mass spectra show non-deuterated peptide, red show the same peptide after unbound (apo) PfRH5FL labeling and blue after PfRH5FL-mAb complex labeling. HDX-MS data for R5.004 were generated following 20 s of labeling and R5.016 after 2 h. (B) 2Fo-Fc electron density maps of the R5.004 and R5.016 variable domains in the bound state, taken from the PfRH5ΔNL:R5.004:R5.016 co-complex structure. The electron density map is contoured at 1.0 σ. (C) Structural alignments of R5.004 and R5.016 Fab fragments unbound (white) and bound to PfRH5ΔNL (R5.004 in blue, R5.016 in red). Light chain and heavy chain CDR loops are annotated.

The binding modes of R5.016 and R5.004, the two most potent known human anti-PfRH5 nAbs, were next determined by X-ray crystallography. A crystal containing PfRH5ΔNL, one R5.016 Fab fragment, and one R5.004 Fab fragment diffracted to a resolution of 4.0 Å (Figure 4A; Table S2). High-resolution structures of unbound R5.016 and R5.004 Fab fragment crystals were also determined, with clear electron density observed for all CDR loops in the bound states, facilitating determination of the complex structure (Figure S4B; Table S2). The R5.004 and R5.016 binding sites were corroborated in solution by hydrogen-deuterium exchange mass spectrometry (HDX-MS) (Figures 4B and S4A). R5.004 binds PfRH5 toward the tip of the “kite-like” structure, contacting the N terminus of helix 4 and each of the three loops that link the converging helices at this apex of PfRH5. These interactions are mediated by five of the CDR loops of the antibody, with only L2 not participating (Table S3). R5.016 binds predominantly to the N terminus of helix 2 of PfRH5. The major contact is mediated by the H3 loop, which lies along the groove between helices 2 and 3. Additional interactions are mediated by the H1, H2, and L2 loops (Table S3). While the CDR loops of R5.004 are not altered upon binding (root-mean-square deviation [RMSD] = 0.58 Å aligning 59/68 CDR α-carbon atoms), four of the CDR loops of R5.016 (H3, L1, L2, and L3) show significant rearrangement upon binding (Figure S4C).

Superimposition of the R5.004 and R5.016 Fab fragment structures on the structure of PfRH5ΔNL:BSG (PDB: 4U0Q) reveals major overlap between the binding sites of BSG and R5.004 (Figure 4C). In contrast, of the two copies of BSG in the PfRH5ΔNL:BSG structure (Wright et al., 2014), one overlaps with R5.016 while one does not. This suggests that, while simultaneous binding is possible, the proximity of R5.016 to the BSG binding site is likely to lead to steric occlusion in the context of an intact IgG antibody and membrane attachment of both components. In comparison to previously studied murine mAbs, R5.016 shares much of its binding site with 9AD4, while R5.004 binds to a distinct epitope which shows some overlap with that of QA1 (Wright et al., 2014). Indeed, 9AD4 and QA1 compete for binding with R5.016 and R5.004, respectively (Figure 3A; Table S1). These data serve to further confirm the BSG binding area and the helical face composed of helices 2 and 3 as two major sites of antibody-mediated neutralization on PfRH5 and identify epitopes for nAbs that are close to human germline and are readily elicited following vaccination.

### Vaccine-Induced mAbs Can Antagonize the Effects of Broadly Neutralizing mAbs

**Figure 5:**
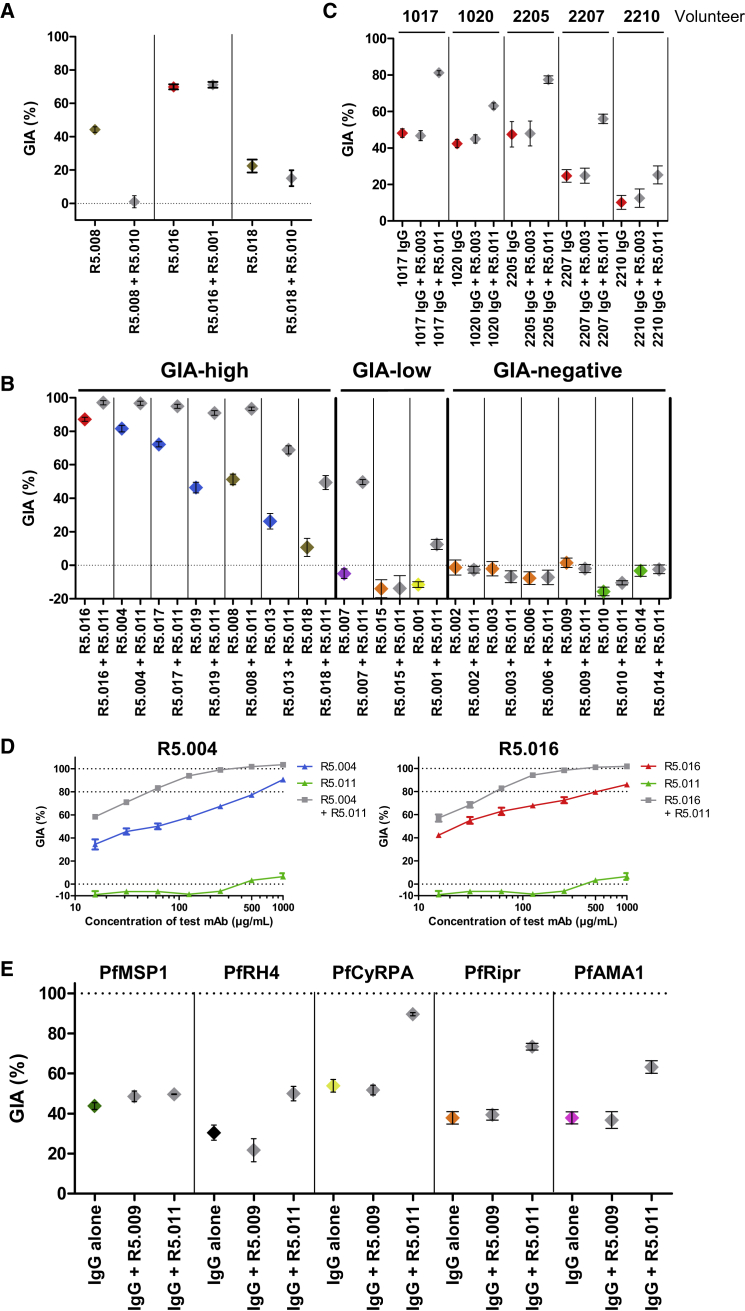
Non-neutralizing mAb R5.011 Potentiates the Growth Inhibitory Effect of Anti-PfRH5 nAbs (A) GIA of nAbs R5.008 (300 μg/mL), R5.016 (150 μg/mL), and R5.018 (400 μg/mL) alone (colored data points) or in the presence of an equimolar concentration of a mAb that blocks its binding to PfRH5FL *in vitro* (gray data points). (B) GIA of human anti-PfRH5 mAbs at 150 μg/mL alone (colored data points) or in the presence of 150 μg/mL R5.011 (gray data points). (C) GIA of total human IgG from five PfRH5FL-vaccinated volunteers (1017, 1020, 2205, 2207, and 2210) at 5 mg/mL, alone (red data points) in the presence of 300 μg/mL of R5.011 or R5.003. (D) GIA of a dilution series of mAbs R5.004, R5.016, and R5.011 alone (blue, red, and green, respectively) as well as a dilution series of the R5.004 and R5.016 nAbs in the presence of an excess of R5.011 (500 μg/mL) (gray) to determine the maximal effect of R5.011. “Concentration of test mAb” refers to the concentration of nAb in mAb combinations. (E) GIA of total IgG from rabbits immunized with PfMSP1 (5 mg/mL, green), PfRH4 (10 mg/mL, black), PfCyRPA (5 mg/mL, yellow), PfRipr (10 mg/mL, orange), or PfAMA1 (3.25 mg/mL, pink) with the addition of 300 μg/mL of R5.011 or R5.009 (gray data points). Concentrations were chosen to achieve ∼30%–60% GIA in the absence of mAb. All data points are the mean of 3 replicates and all error bars show the SEM. Parasites used were 3D7 clone *P. falciparum.* See also Figure S5.

Having identified key neutralizing epitopes on PfRH5, we next assessed the functional activity of combinations of mAbs that bind similar epitopes yet have dissimilar neutralizing properties. We noted that mAbs within the same epitope bin usually cross-compete for binding while those from different epitope bins do not. However, within pairs R5.001/R5.016, R5.008/R5.010, and R5.010/R5.018, mAbs compete for binding to PfRH5 *in vitro* but have differing neutralization properties (Figures 2A, 3B, and S3B; Table S1). We therefore sought to assess the consequence of mAb competition on neutralization. In the case of the R5.008/R5.010 pair, the addition of R5.010 hampered the neutralizing capacity of R5.008 (Figure 5A). However, the same was not true for the R5.001/R5.016 and R5.010/R5.018 pairs, where no significant difference was observed when testing the mixture of two mAbs. These data show that antagonism can occur between mAbs that bind distinct epitope regions on PfRH5, highlighting the need to avoid the production of such antagonistic mAbs during vaccination.

### A Class of Non-neutralizing mAb Potentiates the Effect of Antibodies that Bind PfRH5 and Other Merozoite Proteins

**Figure S5:**
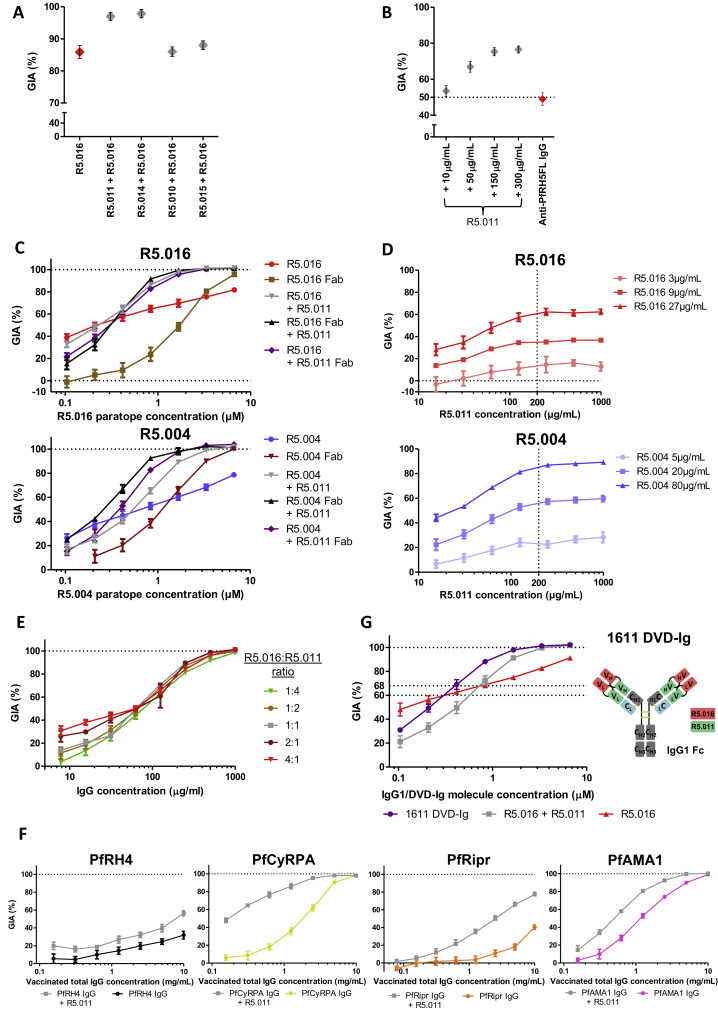
Investigations into the Effect of R5.011, Related to Figure 5 (A) GIA of 150 μg/mL of R5.016 alone or in combination with 150 μg/mL of each mAb from the green epitope bin (and R5.015 as a negative control) to determine whether the potentiating effect of R5.011 occurs with similar mAbs. (B) GIA of polyclonal IgG from a PfRH5FL-vaccinated rabbit at 7 mg/mL alone (red) or mixed with increasing concentrations of R5.011 (gray). (C) GIAs of nAbs R5.004 and R5.016 alone or with R5.011 as various mAb + Fab fragment combinations. x axis concentration values are plotted as the concentration of nAb binding site. mAb-mAb combinations are equimolar and mAb-Fab combinations are equimolar in terms of binding sites. (D) mAb R5.011 is titrated in increasing concentrations into three fixed concentrations of nAb R5.004 or R5.016 to determine the concentration at which R5.011 enhancement reaches a maximum. (E) GIA of titration curves of R5.016 + R5.011 combinations in different molar ratios. (F) GIA of total IgG from a PfRH4-vaccinated rabbit (black), a PfCyRPA-vaccinated rat (yellow), a PfRipr-vaccinated rabbit (orange) and a PfAMA1-vaccinated rabbit (pink) in the presence of an excess of R5.011 mAb (1.5 mg/mL, all gray data points) showing the maximal extent of R5.011-mediated synergy. The x axis shows the concentration of animal-derived IgG only and does not include the R5.011 concentration. (G) GIA of the most potent single human anti-PfRH5 mAb (R5.016), the most potent combination of two human anti-PfRH5 mAbs (R5.016 + R5.011) and of a 1611 bispecific DVD-Ig. The cartoon shows a schematic of the 1611 DVD-Ig comprising R5.011 and R5.016 variable regions. All data points are the mean of 3 replicate wells, all error bars show the SEM. Parasites used were 3D7 clone *P. falciparum*.

Having observed one example of antagonism, we continued our functional assessment of alternative mAb pairs that bind non-overlapping epitopes on PfRH5 and which do not compete for binding. Combinations of mAbs were tested in the GIA assay to determine whether they can improve inhibition of invasion either additively or synergistically (Williams et al., 2012). Remarkably, mAb R5.011 potentiated the inhibitory effect of all eight of the most potent nAbs against 3D7 clone parasites, showing a clear synergistic effect despite itself showing no neutralizing capacity when tested alone. This effect did not extend to GIA-low mAbs R5.001 and R5.015 or any GIA-negative mAbs (Figure 5B and see Figure 2A). R5.011 shares an epitope bin with R5.014 and R5.010 (Figure 3A). R5.014 was also capable of potentiating the effect of a nAb (tested here with R5.016). However, combining R5.010 with R5.016 had no effect (Figure S5A).

The synergistic effect of mAb R5.011 was maintained using either a nAb Fab fragment or a R5.011 Fab fragment, ruling out mechanisms linked to IgG bivalency or the Fc domain (Figure S5C). Synergistic effects were also seen upon addition of R5.011 mAb to polyclonal IgG from PfRH5-vaccinated human volunteers (Figure 5C), as well as rabbits (Figure S5B), indicating that this potentiating phenomenon is far from being maximized in these naturally elicited vaccine-induced IgG.

Dilutions of nAb in the presence of a large excess of R5.011 revealed the maximum possible synergistic effect. Under these conditions, the EC_80_ of R5.016 is reduced from 500 μg/mL to ∼55 μg/mL, and a similar effect was seen for R5.004 (Figure 5D). Furthermore, titrating R5.011 into several fixed concentrations of nAb showed that the maximal potentiating effect is achieved with ∼200 μg/mL of R5.011 (Figure S5D). The optimal ratio of nAb:R5.011 across the concentration range was approximately 4:1, with nAb-biased ratios being more effective at lower concentrations and all ratios roughly equivalent at concentrations above 100 μg/mL (Figure S5E).

We next explored whether the effect of R5.011 was PfRH5-specific by assessing synergy with antibodies targeting merozoite antigens involved in other steps of the RBC invasion process (Weiss et al., 2015, Cowman et al., 2017). The addition of R5.011 mAb to polyclonal IgG from rabbits or rats immunized with PfRH4, PfCyRPA, PfRipr, and PfAMA1 leads to markedly increased GIA, whereas its addition to anti-PfMSP1 IgG had no effect (Figures 5E and S5F). These data suggest R5.011-like antibodies can act synergistically with other functional antibodies targeting the PfRH5 invasion complex antigens as well as other targets such as PfRH4 and PfAMA1, whereas this effect is not observed for antibodies targeting antigens that act prior to merozoite reorientation, such as PfMSP1.

We finally investigated whether R5.016 and R5.011 can be functionally combined into a single molecule by producing a bispecific dual variable domain immunoglobulin (DVD-Ig) containing the variable domains of R5.011 and R5.016. To achieve comparable levels of growth inhibition, a R5.016 + R5.011 mAb combination required fewer molecules than for R5.016 alone (Figure S5G). Furthermore, approximately half the molar concentration of 1611 DVD-Ig is required to achieve the same levels of GIA as the parental R5.016 + R5.011 mAb combination, hinting to a bivalent binding mode of this DVD-Ig molecule. To our knowledge, this DVD-Ig shows the lowest reported EC_80_ for an anti-merozoite antibody-like molecule. Because high levels of merozoite neutralization are known to be required for protection (Douglas et al., 2015, Douglas et al., 2019), the antibody EC_80_ or EC_90_ may prove to be a more useful measure of efficacy than the more widely used EC_50_.

Overall, these data identify R5.011 as a novel antimalarial mAb that displays a new functionality by potentiating the effect of all tested anti-PfRH5 nAbs and pAb, as well as pAb directed against other merozoite antigens, despite having no intrinsic neutralizing properties when tested alone. This raises the critical importance of inducing such antibodies in next-generation PfRH5-based vaccination strategies.

### The R5.011 Epitope Lies at the N Terminus of PfRH5ΔNL

**Figure 6:**
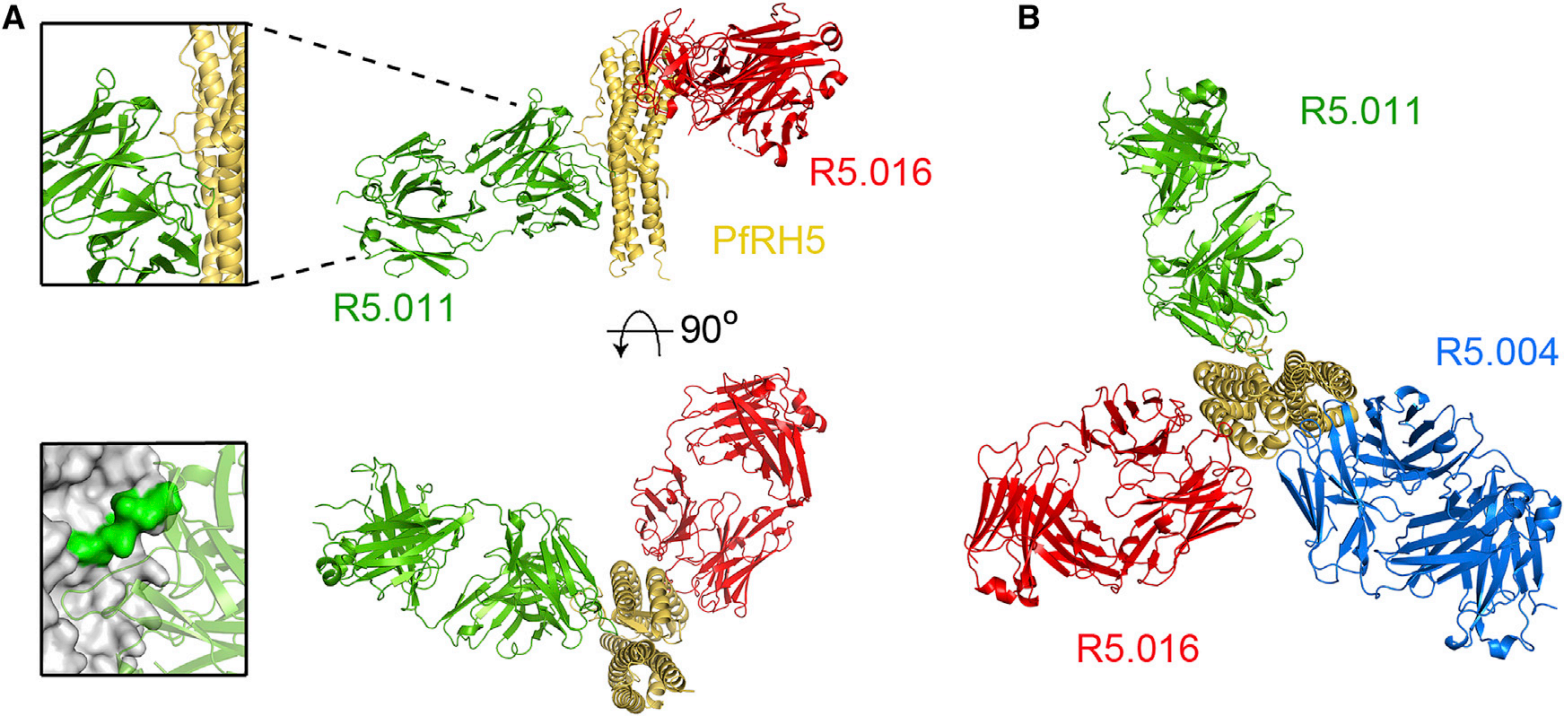
Structure of PfRH5ΔNL in Complex with R5.011 and R5.016 (A) Crystal structure of PfRH5ΔNL bound to Fab fragments from R5.011 and R5.016. The top left inset shows a close-up of the R5.011 epitope. The bottom left inset shows PfRH5 as a gray surface with the peptide most protected by R5.011 mAb in a HDX-MS assay in green. R5.011 is overlaid as a faded cartoon. (B) Overlay of R5.004, R5.011 and R5.016 structures bound to PfRH5ΔNL. See also Figure S6 and Tables S2 and S3.

**Figure S6:**
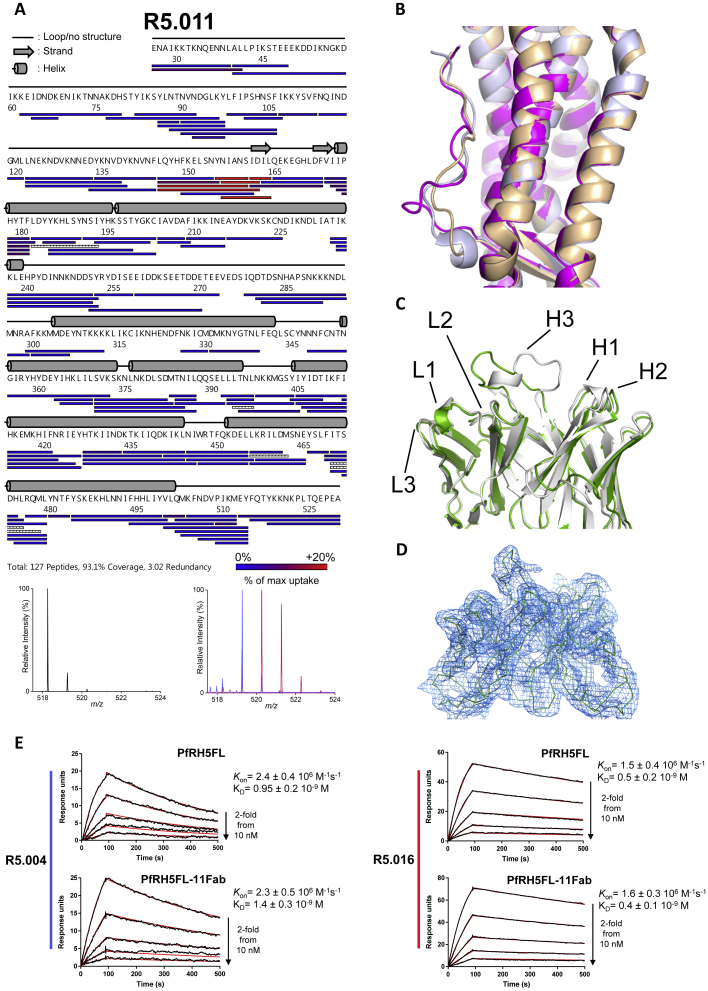
PfRH5 Peptide Protection in HDX-MS by R5.011 and Description of R5.011 Fab Fragment Interaction with PfRH5, Related to Figure 6 (A) Peptide map showing protection of PfRH5FL from deuteration during R5.011 binding. Secondary structure is shown in gray. An example of a highly protected peptide is shown in the mass spectra below (peptide NIANS). Black mass spectra show non-deuterated NIANS peptide, red show the same peptide after unbound (apo) PfRH5FL labeling and blue after PfRH5FL-mAb complex labeling. HDX-MS data for R5.011 was generated following 20 s of labeling. (B) Structural alignment of PfRH5 from the PfRH5ΔNL:R5.004:R5.016 co-complex (in beige), the PfRH5ΔNL:R5.011:R5.016 co-complex (in magenta) and the PfRH5 from PDB entry 4WAT (in light purple), to highlight differences in conformation of the N terminus. (C) Structural alignment of R5.011 Fab fragment unbound (white) and bound to PfRH5ΔNL (green). Light chain and heavy chain CDR loops are annotated. (D) 2Fo-Fc electron density map of the R5.011 variable domains in the bound state, taken from the PfRH5ΔNL:R5.011:R5.016 co-complex structure. The electron density map is contoured at 1.0 σ. (E) SPR sensorgrams of R5.004 (left) or R5.016 (right) binding to PfRH5FL (top) or PfRH5FL-R5.011 Fab fragment complex (bottom). Reported K_D_ and *K*_on_ values are the average of three independent experiments.

To identify the R5.011 epitope, a crystal structure of PfRH5ΔNL bound to one R5.011 Fab fragment and one R5.016 Fab fragment was determined to a resolution of 3.6 Å, with all CDR loops clearly resolved (Figures 6A and S6D). A high-resolution structure of unbound R5.011 Fab fragment was also determined and used, together with structures of R5.016 Fab fragment and PfRH5ΔNL, to provide model restraints (Table S2). R5.011 binds PfRH5 primarily at the interface between the disordered N terminus and the rigid α-helical core at residues Y155–L162, a finding also confirmed by HDX-MS (Figures 6A, lower left inset box, and S6A). Residues F144–N159 of PfRH5 are variably ordered in different crystal structures but are most frequently disordered (Wright et al., 2014, Chen et al., 2014). R5.011 binds to these residues and constrains and orders their conformation as they emerge from the α-helical core of PfRH5 (Figure S6B). All CDR loops except L3 contact PfRH5, with the predominant interaction mediated by H3 (Table S3). Binding is accompanied by a major change in the conformation of the H3 CDR loop of R5.011, which packs against residues Y155–D162 (Figure S6C). The position of R5.016 in PfRH5ΔNL:R5.011:R5.016 is equivalent to that in PfRH5ΔNL:R5.004:R5.016. Figure 6B shows an overlay of R5.004, R5.011 and R5.016 Fab fragments bound to PfRH5ΔNL, radiating in different directions, for comparison. The structures revealed that the binding sites of R5.004 and R5.016 are unaltered (<0.5 Å RMSD aligned over 39 and 30 α-carbon atoms, respectively) by R5.011 binding. Furthermore, the binding affinity and K_on_ of nAbs R5.004 and R5.016 for PfRH5 are unchanged by R5.011 binding (Figure S6E), dismissing structural allostery as a mechanism of anti-PfRH5 nAb potentiation. Thus, R5.011 accesses an epitope largely devoid of secondary structure at a new site on PfRH5.

### R5.011 Potentiates Anti-merozoite nAbs by Increasing the Time Required for Invasion

**Figure 7:**
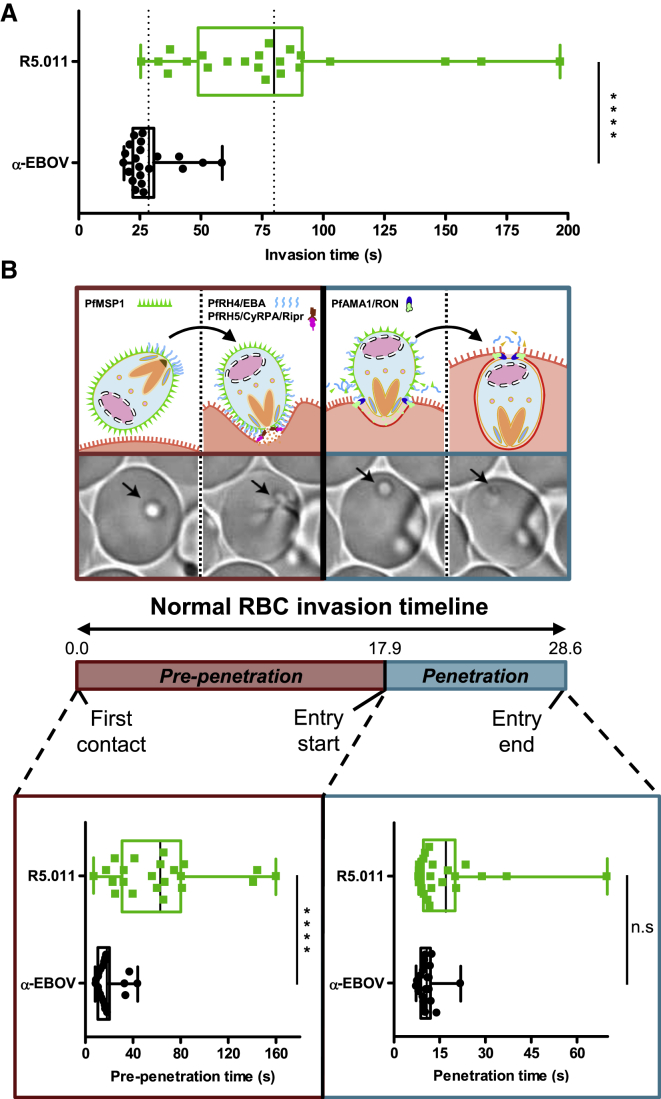
R5.011 Increases Parasite Invasion Time (A) Total time for RBC invasion in the presence of R5.011 or an irrelevant isotype-matched antibody control (α-EBOV). (B) Time for early invasion (pre-penetration) and late invasion (penetration). Data are presented as box-and-whiskers plots showing the interquartile range and total range overlaid on individual data points. Solid black lines show the mean for each group. 3D7 clone *P. falciparum* parasites were used. Both mAbs were used at 500 μg/mL and 21 and 22 invasion events were recorded for α-EBOV and R5.011, respectively. ^∗∗∗∗^p < 0.0001; n.s., non-significant. See also Figure S7 and Videos S1, S2, and S3.

**Figure S7:**
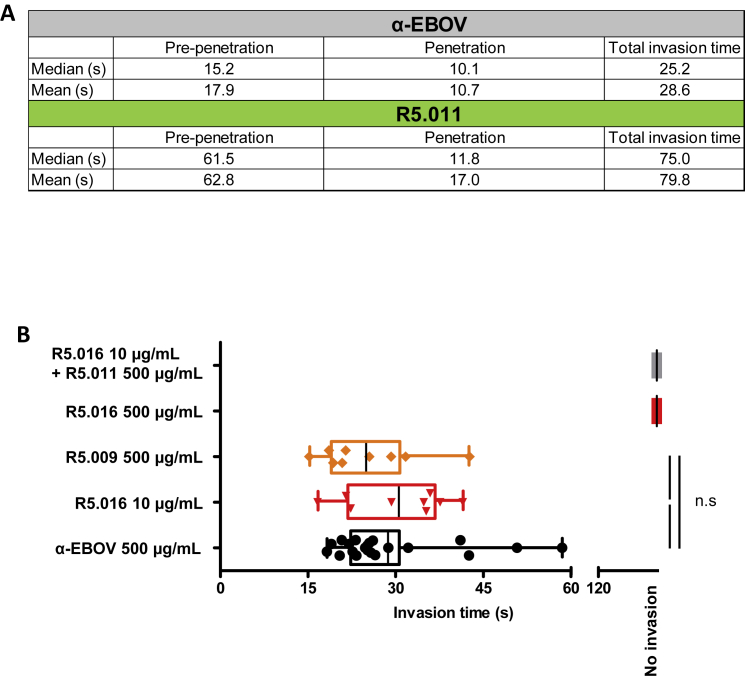
Additional Information from Live-Cell Microscopy Experiments, Related to Figure 7 and Videos S1, S2, and S3 (A) Mean and median early (pre-penetration), late (penetration) and total invasion times of merozoites incubated with 500 μg/mL of R5.011 or α-EBOV. (B) Total invasion times of merozoites in the presence of neutralizing mAb R5.016 (red), synergistic mAb combination R5.011 + R5.016 (gray), non-neutralizing, non-potentiating mAb R5.009 (orange) or α-EBOV (black) mAb at the concentrations indicated. Data are representative of n = 9 invasions for R5.009, R5.016 10 μg/mL and R5.016 + R5.011, n = 12 for R5.016 500 μg/mL, n = 21 for α-EBOV and are presented as box-and-whiskers plots showing the interquartile range and total range overlaid on individual data points. Solid black lines show the mean for each group. 3D7 clone *P. falciparum* parasites were used. n.s = non-significant.

The ability of R5.011 to potentiate the activity of nAbs that bind to various merozoite invasion proteins, together with the demonstration that antibody on-rate is a critical determinant of neutralizing efficacy, led us to hypothesize that this mAb may slow down the invasion process, thus giving longer for the nAbs to reach their binding sites. Indeed, live-cell imaging revealed that the time taken for merozoites to invade RBC is significantly longer (around 3-fold) in the presence of R5.011 versus a control mAb targeting *Ebolavirus* (Figures 7A and S7A; Videos S1 and S2) and versus non-neutralizing, non-potentiating anti-PfRH5 mAb R5.009 (Figure S7B). Concentrations of R5.016 around the EC_50_ did not slow the invasion of those merozoites which were able to invade, whereas high concentrations of R5.016 blocked invasion altogether (Figure S7B; Video S3). The observed delay is largely attributed to the phase of invasion preceding merozoite penetration (Figures 7B and S7A), when invasion ligands PfRH4, PfRH5, PfCyRPA, PfRipr, and PfAMA1 are thought to act. Therefore, R5.011 lengthens the exposure window of critical merozoite targets to nAbs by slowing RBC entry, thus increasing their opportunity to bind and prevent invasion.

Video S1. 3D7 Clone *P. falciparum* Merozoites Invading in the Presence of 500 μg/mL R5.011 mAb, Related to Figures 7 and S7 White arrows denote successful invasions.

Video S2. 3D7 Clone *P. falciparum* Merozoites Invading in the Presence of 500 μg/mL α-EBOV mAb, Related to Figures 7 and S7 White arrows denote successful invasions and black arrows denote unsuccessful invasions.

Video S3. 3D7 Clone *P. falciparum* Merozoites Unsuccessfully Invading in the Presence of 500 μg/mL R5.016 mAb, Related to Figures 7 and S7 Black arrows denote unsuccessful invasions.

## Discussion

Here, we determine the features of a desirable human antibody response to the most advanced blood-stage malaria vaccine candidate antigen, PfRH5, relating the functions of mAbs to their binding sites. We describe different classes of anti-PfRH5 mAb: highly neutralizing mAbs, seemingly inert mAbs, antagonistic mAbs, and a novel class of synergistic non-neutralizing mAbs with the unexpected ability to potentiate the invasion-blocking properties of many nAbs, including those targeting other invasion proteins, by slowing invasion.

Three epitope bins contained GIA-high mAbs: red, blue, and olive. Those in the blue and olive bins function by directly blocking BSG binding, as shown by *in vitro* SPR and AVEXIS assays as well as X-ray crystallography data for mAb R5.004. In contrast, mAb R5.016 (from the more potent red bin) and BSG can simultaneously bind PfRH5 in solution and mAbs from the red epitope bin interfere little with binding to BSG, PfCyRPA, or PfP113. The most likely explanation for the efficacy of R5.016 is the proximity of its binding site on PfRH5 to the BSG binding site. With both PfRH5 and BSG constrained through attachment to their cognate membrane, R5.016 and other similar antibodies are likely to act by preventing PfRH5 from binding to BSG. If so, their improved potency over the BSG-blocking nAbs could be due to increased ease of epitope access on merozoite-bound PfRH5. The fact that antibody association-rate, as opposed to the often-measured affinity, emerged as a key indicator of antibody potency implies that highly dynamic processes are involved in invasion. This is supported by our discovery that a 3-fold lengthening of invasion time correlates with a potentiation of nAb activity. An importance for high antibody on-rates has been noted before for certain viral pathogens (Bates et al., 2014), nevertheless, this is an often-neglected parameter that is highly relevant to blood-stage malaria vaccine design.

In addition, we present our discovery of a novel class of antibody that potentiates the effects of nAbs against various merozoite surface proteins by significantly slowing invasion. This demonstrates potential for wide-ranging synergy and represents a highly novel and attractive means of improving anti-merozoite vaccine-induced nAb efficacy. Cooperativity between pairs of mAbs targeting a single pathogen protein has been described before, whereby two mAbs specific for the same protein display synergistic neutralizing effects. For instance, cooperativity involving non-neutralizing mAbs to *Ebolavirus* glycoprotein and *Neisseria meningitidis* fHBP have been reported (Howell et al., 2017, Beernink et al., 2008). However, to our knowledge, non-neutralizing antibodies like R5.011 that potentiate multiple nAbs against the same and other antigenic targets have not been described for other pathogens. In addition to offering a potential explanation for the surprisingly low GIA EC_50_ of human PfRH5-specific pAb reported previously (Payne et al., 2017), this potentiating phenomenon is an alluring explanation for the synergy reported before between PfRH5 antibodies and those binding other merozoite proteins such as PfRH4 (Williams et al., 2012), PfCyRPA (Reddy et al., 2015), as well as PfMSRP5 and PfRAMA (Bustamante et al., 2017). In addition, if complement neutralization of merozoites acts in the timescale of normal merozoite exposure, as has been argued before (Boyle et al., 2015), extending this time with potentiating antibodies could provide another avenue for synergy with complement.

Notably, we demonstrated there is still substantial room for improvement through adding mAb R5.011 to vaccine-induced human pAb samples, suggesting new avenues to maximize the potency of next-generation PfRH5-based vaccines destined for clinical development. Although relatively high concentrations (around 200 μg/mL) of R5.011 are needed to leverage this effect to its fullest, antigen-specific antibody titers of this magnitude can be achieved in humans (Garçon et al., 2003), however, eliciting them to a small portion of PfRH5 may prove challenging. Delivering potentiating antibody clones alongside a traditional vaccine using vectored immunoprophylaxis (Balazs et al., 2011) could be a more viable alternative. Furthermore, more potent R5.011-like mAbs may be identified in larger sets of anti-PfRH5 mAbs or generated by protein engineering, to achieve maximal effect at lower antibody concentrations. Finally, if one or a combination of R5.004, R5.011, or R5.016 (or closely related mAbs) were used as a therapeutic, natural variation in PfRH5 sequence is unlikely to affect their binding as the only mutations in the vicinity of these contact sites are extremely rare or known not to adversely affect binding.

Overall, the data presented here support the view that vaccine-induced anti-PfRH5 pAb growth inhibition represents a synthesis between the neutralizing effect of nAb and potentiation from R5.011-like antibodies, balanced against antagonism caused by competing antibodies. Immuno-focusing on key epitope regions will be critical for next-generation PfRH5 vaccine design, and the agreement of GIA assay data with *in vivo* protection in NHP (Douglas et al., 2015), as well as identification of anti-PfRH5 mAb clones with *in vivo* efficacy against *P. falciparum* in humanized mice and *Aotus* monkeys (Foquet et al., 2018, Douglas et al., 2019), further underpins this strategy. Antagonism also undoubtedly occurs, through competition between clones binding overlapping epitopes. Furthermore, one can imagine antagonistic antibodies in which the K_on_ of the antagonist is much higher than that of the nAb, leading to a greater effect. The latter type of antibody clone will be detrimental to the overall neutralizing activity of anti-PfRH5 pAb, and thus removal of these epitopes from PfRH5-based vaccine immunogens can only be beneficial. Similarly, there is nothing obvious to gain by inducing the antibodies that appear to have no growth inhibitory or synergistic effects—∼40% of those found in this study.

Our discovery of synergistic and functionally important “non-neutralizing” epitopes challenges the paradigm of structural vaccinology that traditionally sees the advancement of epitopes against which overtly neutralizing antibodies are directed. It is therefore important that future immunogen design strategies for the development of PfRH5-based vaccines use the structural insights obtained here to focus the human immune response to generate both neutralizing and potentiating antibodies. These data thus provide a strategy to design effective PfRH5-based blood-stage malaria vaccines that provide functional anti-merozoite immunity at lower overall concentrations of PfRH5-specific human IgG.

## STAR★Methods

### Key Resources Table

**Table:** 

REAGENT or RESOURCE	SOURCE	IDENTIFIER
**Antibodies**		

c2AC7	Simon J. Draper, Oxford University; Douglas et al., 2019	N/A
c4BA7	Simon J. Draper, Oxford University; Douglas et al., 2019	N/A
c9AD4	Simon J. Draper, Oxford University	N/A
QA1	Simon J. Draper, Oxford University; Douglas et al., 2014	N/A
α-EBOV	Simon J. Draper, Oxford University; Rijal et al., 2019	N/A
Anti-Human IgG (γ-chain specific)−Alkaline Phosphatase antibody produced in goat	Sigma-Aldrich	Cat#A3188; RRID: AB_258057
Anti-Mouse IgG (whole molecule)−Alkaline Phosphatase antibody produced in goat	Sigma-Aldrich	Cat#A9316-.25ML; RRID: AB_258446
Anti-Rabbit IgG (whole molecule)–Alkaline Phosphatase antibody produced in goat	Sigma-Aldrich	Cat#A8025-.5ML; RRID: AB_258372
Rabbit polyclonal anti-PfRH5FL IgG	This paper	N/A
Rabbit polyclonal anti-PfRH5ΔNL IgG	This paper	N/A
Rabbit polyclonal PfAMA1 IgG	Simon J. Draper, Oxford University; Douglas et al., 2011	N/A
Rabbit polyclonal anti-PfRipr IgG	This paper	N/A
Rabbit polyclonal anti-PfRH4 IgG	Simon J. Draper, Oxford University; Douglas et al., 2011	N/A
Rabbit polyclonal anti-PfMSP1 IgG	Simon J. Draper, Oxford University; Douglas et al., 2011	N/A
Rabbit polyclonal anti-PfCyRPA IgG	This paper	N/A
Rat polyclonal anti-PfCyRPA IgG	This paper	N/A

**Bacterial and Virus Strains**		

*Mix & Go* Competent Cells - Strain DH5α *Escherichia coli*	Zymo Research	Cat#T3007
Stellar HST08 strain *Escherichia coli* Competent Cells	Takara	Cat#636763

**Biological Samples**		

PBMC from PfRH5 Phase I trial donors	Simon J. Draper, Oxford University; Payne et al., 2017	N/A
Serum from PfRH5 Phase I trial donors	Simon J. Draper, Oxford University; Payne et al., 2017	N/A
Human O^+^ RBC used in GIA assays	In-house volunteer donations and NHS Blood and Transplant Non-clinical Issue	Cat#NC15-Research Red Cells
Serum from PfRH5-vaccinated rabbits	GenScript	N/A
Serum from PfRH5ΔNL-vaccinated rabbits	GenScript	N/A
Serum from PfAMA1-vaccinated rabbits	Agro-Bio	N/A
Serum from PfRipr-vaccinated rabbit	GenScript	N/A
Serum from PfRH4-vaccinated rabbits	Agro-Bio	N/A
Serum from PfMSP1-vaccinated rabbits	Agro-Bio	N/A
Serum from PfCyRPA-vaccinated rabbits	Cambridge Research Biochemicals	N/A
Serum from PfCyRPA-vaccinated rat	GenScript	N/A
Serum from liver-humanized FRGN mice	This paper	N/A

**Chemicals, Peptides, and Recombinant Proteins**		

PfRipr	This paper	N/A
PfCyRPA	This paper	N/A
PfP113Nt	Gavin J. Wright; Galaway et al., 2017	N/A
PfRH5Nt	Gavin J. Wright; Galaway et al., 2017	N/A
PfRH5FL	Simon J. Draper, Oxford University; Hjerrild et al., 2016	N/A
PfRH5ΔNL	This paper	N/A
PfRH5FL Y147H	This paper	N/A
PfRH5FL H148D	This paper	N/A
PfRH5FL S197Y	This paper	N/A
PfRH5FL C203Y	This paper	N/A
PfRH5FL I410M	This paper	N/A
Basigin	Matthew K. Higgins, University of Oxford; Wright et al., 2014	N/A
RNasin Ribonuclease Inhibitor (Native)	Promega	Cat#N2111
Polyethyleneimine, linear, M.W. 25,000	Alfa Aesar	Cat#43896-03
Borane dimethylamine complex (ABC)	Sigma-Aldrich	Cat#180238-25G
Blocker Casein in PBS	Thermo Fisher Scientific	Cat#37528
SIGMA*FAST* BCIP/NBT alkaline phosphatase substrate	Sigma-Aldrich	Cat#B5655-25TAB
SIGMA*FAST* p-Nitrophenyl phosphate alkaline phosphatase substrate	Sigma-Aldrich	Cat#N1891-50SET
62 biotinylated 20-mer PfRH5FL peptides	Synthesized by Mimotopes, obtained from Simon J. Draper, Oxford University; Payne et al., 2017	N/A
Clophosome-A clodronate liposomes	FormuMax Scientific	Cat#F70101C-A-2
AddaVax vaccine adjuvant	InvivoGen	Cat#vac-adx-10

**Critical Commercial Assays**		

EasySep Human B Cell Enrichment Kit	StemCell Technologies	Cat#19054
Sensiscript RT kit	QIAGEN	Cat#205213
Pierce Fab Preparation Kit	Thermo Fisher Scientific	Cat#44985
JCSG-*plus* crystallization screen	Molecular Dimensions	Cat#MD1-37
NeXtal JCSG Core Suite IV crystallization screen	QIAGEN	Cat#130924
Morpheus crystallization screen	Molecular Dimensions	Cat#MD1-46
Silver bullet crystallization additives	Hampton Research	Cat#HR2-096
ExpiFectamine 293 Transfection Kit	Thermo Fisher Scientific	Cat#A14525

**Deposited Data**		

R5.004 Fab fragment	This paper	PDB: 6RCO
R5.011 Fab fragment	This paper	PDB: 6RCQ
R5.016 Fab fragment	This paper	PDB: 6RCS
PfRH5ΔNL:R5.004:R5.016 complex	This paper	PDB: 6RCU
PfRH5ΔNL:R5.011:R5.016 complex	This paper	PDB: 6RCV

**Experimental Models: Cell Lines**		

Human Expi293F	Thermo Fisher Scientific	Cat#A14527
Human HEK293T	ATCC	Cat#CRL-3216
*Drosophila* S2 cell line expressing PfRH5FL	Simon J. Draper, Oxford University; Hjerrild et al., 2016	N/A
*Drosophila* S2 cell line expressing PfRH5ΔNL	This paper	N/A

**Experimental Models: Organisms/Strains**		

*P. falciparum* 3D7	Carole Long, NIAID	N/A
*P. falciparum* FVO	Carole Long, NIAID	N/A
*P. falciparum* GB4	Carole Long, NIAID	N/A
*P. falciparum* M-Camp	Carole Long, NIAID	N/A
*P. falciparum* Dd2	Carole Long, NIAID	N/A
*P. falciparum* Cp806	Carole Long, NIAID; Williams et al., 2012	N/A
*P. falciparum* Cp845	Carole Long, NIAID; Williams et al., 2012	N/A
*P. falciparum* NF54HT-GFP-luc	Stefan H.I. Kappe; Foquet et al., 2018	N/A
Liver-humanized FRGN KO mice	Yecuris	N/A
Sprague Dawley Rat	GenScript	N/A
New Zealand White rabbits	GenScript, Agro-Bio, Cambridge Research Biochemicals	N/A

**Oligonucleotides**		

Primers for Vγ, Vλ, and Vκ antibody heavy and light chain sequence reverse-transcription and nested PCR	Hedda Wardemann; Tiller et al., 2008	N/A
Primers for amplifying and cloning human Vγ, Vλ, and Vκ antibody heavy and light chain sequences	This paper	N/A

**Recombinant DNA**		

AbVec-hIg expression plasmids	Patrick C. Wilson, University of Chicago; Wrammert et al., 2008	N/A
pExpreS^2^-1 *Drosophila* S2 expression plasmid	Simon J. Draper, Oxford University; Hjerrild et al., 2016	N/A

**Software and Algorithms**		

XDS	http://xds.mpimf-heidelberg.mpg.de/	RRID: SCR_015652
Phaser	https://www.phenix-online.org/documentation/reference/phaser.html	RRID: SCR_014219
BUSTER	https://www.globalphasing.com/buster/	RRID: SCR_015653
PHENIX	http://www.phenix-online.org	RRID: SCR_014224
COOT	https://www2.mrc-lmb.cam.ac.uk/personal/pemsley/coot/	RRID: SCR_014222
PyMOL version 2.3.0	Schrödinger (https://pymol.org/2/)	RRID: SCR_000305
GraphPad Prism version 5-7	GraphPad (https://www.graphpad.com/)	RRID: SCR_002798
IgBLAST	https://www.ncbi.nlm.nih.gov/igblast/	RRID: SCR_002873

**Other**		

Octet Biosensor / Streptavidin (SA)	FortéBio	Cat#18-5019
Biacore Biotin CAPture Kit	GE Healthcare	Cat#28920233
Biacore Sensor Chip CM5	GE Healthcare	Cat#BR-1000-12
Biacore Sensor Chip Protein A	GE Healthcare	Cat#29127558

### Lead Contact and Materials Availability

Further information and requests for resources should be directed to and will be fulfilled by the Lead Contact, Simon J. Draper (simon.draper@ndm.ox.ac.uk).

### Experimental Model and Subject Details

#### Human blood sample collection

Healthy, malaria-naive males and non-pregnant females aged 18-50 were invited to participate in the VAC057 study of the PfRH5-based vaccine (Payne et al., 2017). VAC057 was a first-in-human, open-label, non-randomized, dose escalation Phase Ia clinical trial evaluating the safety and immunogenicity of the viral vectored vaccines ChAd63 RH5 and MVA RH5 in a heterologous prime-boost regime with an eight week interval. The study was conducted in the UK at the Centre for Clinical Vaccinology and Tropical Medicine (CCVTM), University of Oxford, Oxford, and the NIHR Wellcome Trust Clinical Research Facility (WTCRF) in Southampton. The study received ethical approval from the Oxfordshire Research Ethics Committee A in the UK (REC reference 14/SC/0120). The study was also reviewed and approved by the UK Medicines and Healthcare products Regulatory Agency (MHRA, reference 21584/0331/001-0001). Volunteers signed written consent forms and consent was verified before each vaccination. The trial was registered on Clinicaltrials.gov (NCT02181088) and was conducted according to the principles of the current revision of the Declaration of Helsinki 2008 and in full conformity with the ICH guidelines for Good Clinical Practice (GCP). The primary endpoint of the study was to assess the safety of ChAd63 RH5 and MVA RH5, with a secondary endpoint to assess immunogenicity. Human blood samples were collected into lithium heparin-treated vacutainer blood collection systems (Becton Dickinson). PBMC were isolated and used within 6 hours in fresh assays, otherwise excess cells were frozen in fetal calf serum (FCS) containing 10% dimethyl sulfoxide and stored in liquid nitrogen. Plasma samples were stored at −80°C. For serum preparation, untreated blood samples were stored at room temperature and then the clotted blood was centrifuged for 5 min at 1000*g*. Serum was stored at −80°C.

#### Experimental animal models

Sprague Dawley rat and female New Zealand White rabbit immunizations (PfRH5FL, PfRH5ΔNL and PfRipr) were carried out by Genscript (Piscataway, NJ, USA). GenScript holds a valid and current Animal Welfare Assurance in compliance with the Public Health Service (PHS) Policy on humane Care and Use of Laboratory Animals as granted by the Office of Laboratory Animal Welfare (OLAW). Female New Zealand White rabbit immunizations (PfCyRPA) were carried out by Cambridge Research Biochemicals (Billingham, UK) in compliance with the UK Animals (Scientific Procedures) 1986 Act (ASPA). Female New Zealand White rabbit immunizations (PfRH4, PfMSP1 and PfAMA1) were carried out by Agro-Bio (La Ferté Saint Aubin, France) according to the current French version of the European Directive 2010/63/EU and following a protocol approved by their internal ethics committee. The study using liver-humanized FRGN KO mice was carried out in accordance with the recommendations of the NIH Office of Laboratory Animal Welfare standards (OLAW welfare assurance # A3640-01). The protocol was approved by the Center for Infectious Disease Research Institutional Animal Care and Use Committee (IACUC) under protocol SK-16.

#### Cell lines

Adherent HEK293T cells were grown in Dulbecco’s Modified Eagle Medium (DMEM) (Sigma-Aldrich) supplemented with 2 mM L-glutamine, 1 mM sodium pyruvate, 100 U/mL penicillin, 0.1 mg/mL streptomycin and 10% ultra-low IgG fetal bovine serum (FBS) (all from Thermo Fisher Scientific) in a static incubator at 37°C, 8% CO_2_. Expi293F HEK cells were cultured in suspension in Expi293 expression medium (Thermo Fisher Scientific) at 37°C, 8% CO_2_, on an orbital shaker set at 125 RPM. *Drosophila* S2 were cultured in suspension in EX-CELL 420 medium (Sigma-Aldrich) supplemented with 100 U/mL penicillin, 0.1 mg/mL streptomycin and 10% FBS at 25°C.

### Method Details

#### Generation of monoclonal antibodies

##### Plasmablast isolation and sorting

Volunteers from a Phase Ia safety and immunogenicity clinical trial were bled seven days after the second immunization using MVA (modified vaccinia virus Ankara) encoding PfRH5FL (Payne et al., 2017). Blood was collected from volunteers in heparinized tubes and centrifuged in Leucosep tubes (Greiner Bio one) to separate the peripheral blood mononuclear cells (PBMC). The PBMC were enriched for B cells using a human pan-B cell enrichment kit (StemCell Technologies, Inc.) and resuspended in DMEM before staining with a CD19^+^, CD10^–^, CD21^–^, CD27^+^, CD20^–^, CD38^+^, IgG^+^ fluorophore-conjugated antibody panel. Plasmablasts were single-cell sorted using a MoFlo cell sorter (Dako cytomation) into 96-well PCR plates containing 10 μL of 10 mM Tris HCl buffer containing 40 U/mL of RNase inhibitor (Promega). The study received ethical approval from the Oxfordshire Research Ethics Committee A in the UK (REC reference 14/SC/0120). The volunteers signed written consent forms and consent was verified before each vaccination.

##### Antibody variable gene amplification

In each well of a 96-well plate containing a single antibody-secreting cell (ASC), a two-step RT-PCR was carried out with a first reverse transcription (RT) step using a Sensiscript RT kit (QIAGEN) and degenerate primers 1-17 (modified from Tiller et al. [2008]) (see Table S4). Next, a first PCR (PCR1) was performed on 1 μL of the RT reaction product using the same set of primers used before (1-17) which cover the diversity of all Vγ, Vκ and Vλ sequences using Phusion HF master mix (New England Biolabs). Following this, a second PCR (PCR2) was performed using primers 18-51 (Table S4), also using Phusion HF master mix, on 1 μL of the previous product diluted 1:100 to amplify inserts which contain plasmid-homologous extensions designed for circular polymerase extension cloning (CPEC) (Quan and Tian, 2009).

##### Cloning

The AbVec-hIgG1/AbVec-hIgKappa/AbVec-hIgLambda expression plasmids were a kind gift from Patrick C. Wilson (University of Chicago) (Wrammert et al., 2008). These plasmids were 5′ digested using BshTI and at the 3′ using SalI (AbVec-hIgG1), XhoI (AbVec-hIgLambda) and Pfl23II (AbVec-hIgKappa) to yield linear products. CPEC assembly was done by mixing 100 ng of a 1:1 molar ratio of insert:plasmid in 20 μL containing 1x Phusion HF polymerase master mix and assembled using an 8-cycle CPEC protocol (8 cycles: 98°C 10 s, slow ramp anneal 70°C → 55°C at 0.1°C/s, 72°C 35 s). Full nicked plasmids were subsequently transformed into Zymo 5α Mix & go competent *Escherichia coli* (Zymo Research) according to manufacturer’s instructions, streaked on LB agar Petri dishes containing 100 μg/mL carbenicillin and grown at 37°C overnight in a static incubator. Colonies were screened by PCR for inserts of the correct size.

##### Screening

Exponential growth-phase adherent HEK293T cells were resuspended in DMEM (Sigma-Aldrich) supplemented with 2 mM L-glutamine, 1 mM sodium pyruvate, 100 U/mL penicillin, 0.1 mg/mL streptomycin and 10% ultra-low IgG FBS (all from Thermo Fisher Scientific) and seeded at 4 × 10^4^ cells/well in 100 μL 24 h prior to transfection in Costar 96-well cell culture plates (Corning). On the day of transfection, for each well, 50 μL of 60 μg/mL linear 25 kDa PEI (Alfa Aesar) was mixed with 200 ng of cognate heavy- and light-chain coding plasmid in a volume of 50 μL and shaken at 20°C for 30 min. The DNA-PEI complexes were then added to the HEK293T cells. The next day, an additional 50 μL of supplemented DMEM (as described above) was added to each well. Supernatants were screened for PfRH5FL binding by indirect ELISA as described in the ELISA methods section.

#### Protein expression and purification

The recombinant PfRH5 sequence used in all experiments except for those involving BLI (Figures 1C, 3A, S3A, and S3B; Table S1) was based on the 3D7 clone *P. falciparum* reference sequence and encoded amino acids E26-Q526. The sequence also encoded a C-terminal four-amino acid purification tag (C-tag: EPEA) and four mutations to delete N-linked glycosylation sequons (T40A, T216A, T286A and T299A) and was named “PfRH5FL” (this recombinant protein is also known as “RH5.1”) (Jin et al., 2018). This protein was expressed as secreted protein by a stable monoclonal *Drosophila* S2 cell line (Hjerrild et al., 2016) and affinity purified using CaptureSelect C-tag affinity matrix (Jin et al., 2017) (Thermo Fisher Scientific). A further size-exclusion chromatography (SEC) polishing step was done on a HiLoad 16/60 Superdex 200 pg column (GE Healthcare) to separate monomers from oligomers and contaminants as well as to buffer-exchange the protein into 20 mM Tris, 150 mM NaCl, pH 7.5. A detailed description of the production of PfRH5FL was described by Jin et al., 2018. The recombinant PfRH5 sequence (also named “PfRH5FL” for simplicity) used in BLI experiments also encoded amino acids E26-Q526 of the 3D7 clone *P. falciparum* reference sequence, with only two mutations to delete N-linked glycosylation (N38Q and N214Q). This sequence is identical to the vaccine sequence (Payne et al., 2017) and was expressed with an additional C-terminal AviTag and Strep-II® tag in tandem. This protein was expressed in Expi293F HEK cells as a secreted, monobiotinylated protein as described previously by Bushell et al. (2008).

The recombinant PfRH5ΔNL sequence used was based on the 3D7 clone *P. falciparum* reference sequence and encoded amino acids K140-K247 and N297-Q526 with two mutations to delete N-linked glycosylation sequons (T216A and T299A) and with the addition of a C-terminal C-tag. PfRH5ΔNL was expressed as secreted protein from stably transfected polyclonal *Drosophila* S2 cells. Its purification is detailed in the X-ray crystallography experimental procedures section.

The PfRipr expression construct used for rabbit vaccination to yield IgG used in Figures 5E and S5F comprised amino acids M1-N1086 with eight mutations introduced to ablate N-linked glycosylation (N103Q, N144Q, N228Q, N334Q, N480Q, N498Q, N506Q, N526Q, N646Q, N647Q, N964Q and N1021Q) followed by a C-terminal C-tag. PfRipr was purified by C-tag affinity chromatography followed by SEC as detailed above for PfRH5. The expression construct used for monomeric PfCyRPA production in Figure 3C and rat vaccination in Figure S5F was based on the 3D7 clone *P. falciparum* sequence and comprised amino acids D29-E362 with three mutations introduced to ablate N-linked glycosylation (S147A, T324A and T340A) and also included a C-terminal GGGS linker followed by a 4-amino acid C-tag (EPEA). The PfCyRPA immunogen sequence used for rabbit vaccination in Figure 5E was identical to that described above with the C-tag replaced by a C-terminal CD4 tag comprising rat domains 3 and 4 (CD4d3+4) tag followed by a hexahistidine (His6) tag. The protein was expressed as secreted protein from Expi293F HEK cells and purified by C-tag affinity chromatography followed by SEC on a HiLoad 16/60 Superdex 200 pg column (GE Healthcare). Basigin protein comprised of immunoglobulin domains 1 and 2 of the short isoform (residues A22-H205) and was expressed from *E. coli* and purified by Ni^2+^-affinity and size exclusion chromatography. Further details can be found in Wright et al., 2014. PfP113Nt, encoding amino acids Y23-K219 of PfP113 (3D7) and PfRH5Nt encoding amino acids F25-K140 of PfRH5 (3D7), were expressed encoding C-terminal tags comprising CD4d3+4, a biotin acceptor peptide and a His6 tag in tandem. For further details see Galaway et al. (2017).

Recombinant monoclonal antibodies were transiently expressed in Expi293F HEK cells. Cognate heavy and light chain-coding plasmids were co-transfected at a 1:1 ratio. Supernatants were harvested by centrifuging the culture at 2500 x*g* for 15 min and filtering the supernatant with a 0.22 μm vacuum filter. All mAbs were purified using a 5 mL Protein G HP column (GE Healthcare) on an ÄKTA start FPLC system or an ÄKTA Pure FPLC system (both GE Healthcare). Equilibration and wash steps were performed with Dulbecco’s PBS and mAbs were eluted in 0.1 M glycine pH 2.7. The eluates were pH equilibrated to 7.4 using 1.0 M Tris HCl pH 9.0 and immediately buffer-exchanged into Dulbecco’s PBS and concentrated using an Amicon^®^ ultra centrifugal concentrator (Millipore) with a molecular weight cut-off of 30 kDa. IgG from serum in Figures 3E, 3F, 3H, 5C, 5E, S5B, and S5F was purified on drip columns packed with Pierce Protein G agarose resin (Thermo Fisher Scientific). Pierce protein G IgG binding buffer (Thermo Fisher Scientific) was used to dilute the serum 1:1 before loading as well as for equilibration and wash steps. Bound IgG was subsequently eluted, neutralized and concentrated as above. Bispecific DVD-Ig 1611 was constructed by cloning R5.016 variable regions upstream (5′) of R5.011 variable regions, separated by ASTKGPSVFPLAP and TVAAPSVFIFPP linkers for the heavy and light chains, respectively. 1611 DVD-Ig expression and purification was conducted as described above for monospecific mAbs.

#### X-Ray crystallography

##### Complex preparation

PfRH5ΔNL was purified from *Drosophila* S2 culture supernatant using C-tag affinity chromatography and glycosylated contaminants were removed by a subsequent lectin chromatography step with a HiTrap ConA 4B column (GE Healthcare). Disordered regions were trimmed by an overnight incubation at 20°C with endoproteinase gluC (New England Biolabs) at a final concentration of 1 μg/mL. Fab fragments were generated by papain digestion using a Pierce Fab Preparation Kit (Thermo Fisher Scientific) following the manufacturer’s recommendations. Complexes were prepared by mixing each Fab fragment with PfRH5ΔNL at a 1:1 molar ratio and were methylated with 1 M ABC (Borane dimethylamine complex) and 1 M formaldehyde (both Sigma-Aldrich) (Walter et al., 2006). The methylated complexes were subjected to SEC on a HiLoad 16/60 Superdex 200 pg column (GE Healthcare) at 4°C in 20 mM Tris pH 7.4, 150 mM NaCl. The complex-containing fractions were pooled and concentrated using an Amicon^®^ ultra centrifugal concentrator (Millipore) with a molecular weight cut-off of 30 kDa.

##### Crystallization, data collection and processing

Crystallization was achieved using vapor diffusion in sitting drops. Crystals were obtained for the Fab fragments of mAbs R5.004, R5.011 and R5.016 alone, as well as for complexes consisting of PfRH5ΔNL:R5.004:R5.016 and PfRH5ΔNL:R5.011:R5.016. In each case, a TTP Labtech Mosquito LCP robot was employed to mix 100 nL of each protein complex at a concentration of 10 mg/mL with 100 nL of well solutions from commercially available crystal screens.

Crystals of R5.004 Fab fragments were obtained in the JCSG-plus crystallization screen (Molecular Dimensions) and were optimized with a final well solution of 0.2 M lithium sulfate, 0.1 M NaAc pH 4.5, 30% PEG 8000 and 0.06% hexamminecobalt(III) chloride, 12 mM MES monohydrate, 12 mM PIPES, 4 mM HEPES chloride (Silver bullet additive D3, Hampton Research). They were cryo-protected by transfer into well solution supplemented with 25% glycerol, then cryo-cooled by plunging into liquid nitrogen. Data were collected on beamline I-03 at Diamond Light Source (Harwell, UK), leading to a complete dataset at a resolution of 1.7 Å.

Crystals of R5.016 Fab fragments were obtained in the JCSG-plus crystallization screen (Molecular Dimensions), with a final well solution of 0.2 M lithium sulfate, 0.1 M NaAc pH 4.5, 50% PEG 400. Crystals were cryo-cooled directly in the well solution by plunging into liquid nitrogen. Data were collected on beamline I04-1 at Diamond Light Source (Harwell, UK), leading to a complete dataset at a resolution of 2.1 Å.

Crystals of R5.011 Fab fragments were obtained in the NeXtal JCSG-IV crystallization screen (QIAGEN), with a final well solution of 0.16 M ZnAc, 0.108 M Na cacodylate pH 6.5, 14.4% PEG 8000, 20% glycerol. They were cryo-protected by transfer into well solution supplemented with 25% glycerol, then cryo-cooled by plunging into liquid nitrogen. Data were collected on beamline PROXIMA-1 at SOLEIL (Saint-Aubin, France), leading to a complete dataset at a resolution of 2.3 Å.

Crystals of the PfRH5ΔNL:R5.004:R5.016 complex were obtained in the JCSG-plus crystallization screen (Molecular Dimensions), with a final well solution of 0.15 M DL malic acid, 20% PEG 3350. They were cryo-protected by transfer into well solution supplemented with 25% glycerol, then cryo-cooled by plunging into liquid nitrogen. Data were collected on beamline I04 at Diamond Light Source (Harwell, UK), leading to a complete dataset at a resolution of 4.0 Å.

Crystals of the PfRH5ΔNL:R5.011:R5.016 complex were obtained in the Morpheus crystallization screen (Molecular Dimensions), with a final well solution of 10% PEG 20000, 20% PEG 550MME, 0.02 M amino acids, 0.1 M MES/imidazole pH 6.5. Crystals were cryo-cooled directly in the well solution by plunging into liquid nitrogen. Data were collected on beamline ID23 at the European Synchrotron Radiation Facility (Grenoble, France), leading to a complete dataset at a resolution of 3.6 Ε.

In each case, data reduction was performed using XDS (Kabsch, 2010). Molecular replacement solutions were found for each individual Fab fragment, using Phaser (McCoy et al., 2007) with the most closely related Fab fragment structure in the PDB, split into their constant and variable domains, as search models (4KQ3 for R5.004, 4HK0 for R5.011 and 5K9O for R5.016). This led to a cycle of model building and refinement using COOT (Emsley et al., 2010) and BUSTER (Bricogne et al., 2011). For R5.004, this resulted in a complete model for the Fab fragment. For R5.011, this resulted in a complete model for the Fab fragment, with the exception of residues 143-148 in the heavy chain, which were disordered in the electron density map. For R5.016, this resulted in a complete model for the Fab fragment, with the exception of residues 103-108 in the heavy chain and 1-7, 25-29 and 55-58 in the light chain, all of which were disordered in the electron density map.

The structure of the PfRH5ΔNL:R5.011:R5.016 complex was obtained to 3.6 Å resolution using Phaser with the structure of PfRH5ΔNL (PDB code: 4U0R) and those of Fab fragments obtained above, split into their constant and variable domains, as search models. All domains were well resolved, although significant changes in conformation were observed in CDR H3 of both R5.011 and R5.016, as a result of PfRH5ΔNL binding. COOT, BUSTER and PHENIX (Afonine et al., 2012) were used for refinement, including application of restraints that derived from the higher resolution structures of PfRH5ΔNL and the shared regions of the Fab fragments. This allowed the production of a final model in which all of the Fab fragment domains, and all of the CDR loops, were clearly resolved.

The structure of the PfRH5ΔNL:R5.004:R5.016 complex was obtained at 4 Å resolution, using Phaser. The structure of PfRH5ΔNL bound to the variable domain of R5.016, obtained from the structure of the PfRH5ΔNL:R5.011:R5.016 complex, and the structure of the variable domain of R5.004 obtained above, were used as search models in Phaser. No significant changes were observed in the CDR loops of the Fab fragment of R5.004. Placement of the constant domains of R5.016 and R5.004 was challenging, due to anisotropy resulting from disorder within the crystal. These domains were therefore placed using real space docking after refinement in BUSTER of a structure consisting of PfRH5ΔNL and the variable domains of R5.004 and R5.016. This gave unambiguous electron density for the regions of the constant domains which lie close to the contact with the variable domains, but, after refinement, electron density is still absent for the PfRH5ΔNL-distal parts of the constant domains. Refinement in COOT, BUSTER and PHENIX allowed the production of a final model in which the Fab fragment domains, and all of the CDR loops, were clearly resolved.

#### Humanized mouse passive transfer

##### P. falciparum sporozoite production and mouse infection

The luciferase expressing strain *P. falciparum* NF54HT-GFP-luc was maintained in RPMI 1640 supplemented with 25 mM HEPES, 2 mM L-glutamine, 50 μM hypoxanthine, 10% human serum and sub-cultured in 5% human group O RhD positive human RBC. Briefly, asexual cultures were inoculated at 1% parasitemia with no further sub-culturing and daily media changes to induce gametocytogenesis. Mature gametocytes were fed to 4-day old *Anopheles stephensi* mosquitoes to initiate infection. Mosquitoes were incubated at 27°C and 75% humidity for 14 d and given 8% dextrose + PABA to foster parasite growth. Liver humanized FRGN KO mice were purchased from Yecuris Corp. and showed human hepatocyte repopulation levels above 70% determined by human serum albumin levels. Liver humanized mice were cycled on NTBC once a month for 3 d at 8 μg/mL in the drinking water to maintain health. Animals did not receive NTBC three weeks prior to and during the infection study. For mosquito bite infection, 3-5 liver humanized FRGN KO mice were anesthetised and placed on top of a mosquito cage containing 150-250 infected mosquitoes for 20 min.

##### P. falciparum liver-to-blood stage transition

On the day of challenge, liver humanized FRGN KO mice were injected both in the retro-orbital plexus and the peritoneal cavity with 50 μL clodronate-containing liposomes (Clophosome®-A, FormuMax Scientific), 100 mg/kg cyclophosphamide (Sigma-Aldrich). Five days after challenge, the animals were bled a volume of approximately 200 μL, and 500 μL hRBC was injected in the retro-orbital plexus (70% O+ human erythrocytes in RPMI 1640 supplemented with 25 mM HEPES, 2 mM L-glutamine, 50 μM hypoxanthine and 10% human serum). The next day, mice were bled 200 μL and received an intraperitoneal injection of 700 μL hRBC. mAb transfer was done on day 6 to precede the establishment of blood-stage infection. Dosage was 15 mg total rabbit IgG per mouse formulated in PBS and delivered intravenously. The clodronate liposome and cyclophosphamide injections were repeated on days 5, 9, 11, 13. Human RBC were injected daily in a volume of 300-700 μL to keep the percentage of hRBC stable around 50%–60%. If the percentage reached more than 70%, the mice were not injected with hRBC to limit morbidity. On days 9, 11 and 13 each mouse was bled from the retro-orbital plexus to sample antibody levels.

##### Quantification of parasite burden

Luciferase activity was measured in the mice using the IVIS Lumina II animal imager (Perkin Elmer). The abdomen of mice was shaved to enhance detection. Mice were injected intraperitoneally with 100 μL of luciferase substrate RediJect D-Luciferin (Perkin Elmer) to quantitate specific enzymatic activity. Animals were anesthetized and imaged within 5 min of substrate injection. Signal was acquired for 5 min using a field of view of 10 cm and medium binning factor. Living Image 3.0 software was used to measure total flux (photons/second) of a region of interest, which was placed around each mouse. A comprehensive description of the mouse model was published by Foquet et al., 2018.

##### PfCyRPA/PfP113/BSG SPR blocking assays

Data were collected on a Biacore X100 (GE Healthcare). All data used were reference subtracted from a non-immobilized flow cell (Fc 2-1). Binding values were measured manually in the Biacore X100 control software.

##### PfRH5-basigin binding

Experiments were performed at 25°C in SPR running buffer (PBS + 0.005% Polysorbate-20, GE Healthcare). Approximately 670 RU of basigin was amine-coupled to a CM5 chip (GE Healthcare) on flow cell 2 (Fc 2) using standard NHS/EDC chemistry. PfRH5FL-mAb complexes were made by mixing PfRH5FL and mAb to a final concentration of 0.5 μM PfRH5FL and 1 μM mAb in SPR running buffer. Complexes were injected over Fc 1 and Fc 2 at a flow rate of 10 μL/min for 30 s and the surface was regenerated with 10 mM glycine pH 1.5 for at a flow rate of 10 μL/min for 60 s. Between experiments, one injection of 0.5 μM PfRH5FL was made to assess basigin degradation caused by regeneration.

##### PfRH5-PfCyRPA binding

Experiments were performed at 25°C in SPR running buffer (PBS + 0.005% Polysorbate-20, GE Healthcare) using a sensor chip protein A (GE Healthcare). mAb was injected at a concentration of 20 nM at a flow rate of 5 μL/min for 35 s over Fc 2 on a protein A coated chip (GE Healthcare). Next, PfRH5FL was injected over Fc 1 and Fc 2 at a concentration of 50 nM at a flow rate of 10 μL/min for 120 s before injecting PfCyRPA at a concentration of 1 μM for 120 s, also at a flow rate of 10 μL/min. The chip surface was regenerated with 10 mM glycine pH 1.5 for at a flow rate of 10 μL/min for 60 s.

##### PfRH5-PfP113 binding

Experiments were performed at 37°C in SPR running buffer (PBS + 0.005% Polysorbate-20, GE Healthcare) using a Sensor chip CAP (GE Healthcare). The whole experiment was run at a flow rate of 5 μL/min. Approximately 1500 RU of CAP reagent (GE Healthcare) was captured on Fc 1 and Fc 2. On Fc 1, approximately 500 RU of a biotinylated control CD4d3+4-tagged protein was immobilized. On Fc 2, each experiment consisted of capturing 1500 RU of CAP reagent in an 80 s injection followed by approximately 1800 RU of PfP113Nt in an 80 s injection at a concentration of 20 μg/mL. After this, PfRH5FL-mAb complex (both at 1 μM) was flowed over Fc 1 and Fc 2. Flow cell 2 was regenerated between experiments in a 110 s injection of 6 M guanidine + 250 mM NaOH, as per the manufacturer’s instructions.

#### Bio-Layer Interferometry

All BLI was carried out on an OctetRED384 (FortéBio) using streptavidin-coated biosensors (FortéBio) to immobilize PfRH5FL enzymatically monobiotinylated on a C-terminal AviTag. Assays were carried out in 96-well format in black plates (Greiner). For assaying mAb binding to PfRH5FL variants (Figure 1C), the experiment followed a four-step sequential assay: Baseline (PBS, 30 s); Protein immobilization (neat supernatant, 180 s); Wash (PBS, 60 s); and mAb binding (150 nM mAb, 120 s). Graphed data show the fold-change in binding of each mAb to the 3D7 PfRH5FL reference protein relative to the binding of each mAb to each mutant protein after correction for PfRH5FL immobilization level on each biosensor. The fold-change values which were inferior to 1 were plotted as their inverse to avoid skewing in their data representation compared to fold-change values greater than 1. For epitope binning studies in Figures 3A and S3A and Table S1 the experiment followed a six-step sequential assay: Baseline (PBS, 30 s); Protein immobilization (neat supernatant, 120 s); Wash (PBS, 60 s); mAb1 binding (300 nM mAb1, 120 s); Wash (PBS, 60 s); mAb2 binding (150 nM mAb2, 120 s). “Relative binding” shown in Table S1 shows the ratio (Signal_mAb2_ with mAb1 bound)/(Signal_mAb2_ with no mAb1) where “Signal_mAb2_” was normalized for the amount of PfRH5FL bound to the biosensor such that “Signal_mAb2_” = the raw signal in “mAb2 binding” divided by the raw signal in the “Protein immobilization” phase. The resulting “binding profile” for any given mAb corresponds to the column of “relative binding values” under that mAb in the “relative binding” table. To establish the epitope bins, binding profiles between each mAb pair was correlated using a person product-moment correlation coefficient, the values of which are shown in the “binding profile correlation” table in Table S1. mAb pairs whose binding profile correlation was > 0.7 were grouped into the same epitope bin.

#### ELISA

Qualitative mAb binding ELISAs such as those used in Figures 1D, 3D, and S3F were carried out by coating PfRH5FL, PfRH5ΔNL or PfRH5Nt on Maxisorp flat-bottom 96-well ELISA plates (Nunc) at 2 μg/mL in 50 μL at 4°C overnight. In Figure 1D, PfRH5FL was heat-treated by incubation at 90°C for 10 min. Plates were then washed twice with PBS-Tween 20 and blocked with 200 μL of Blocker Casein (Thermo Fisher Scientific) for 1 h. Next, wells were incubated with 1000 ng/mL of mAb for approximately 45 min at 20°C then washed 4 times with PBS-Tween 20 before the addition of 50 μL of goat anti-human gamma-chain alkaline phosphatase-conjugated secondary antibody (goat anti-mouse IgG alkaline phosphatase-conjugated secondary antibody for QA1) (both Sigma-Aldrich) for 45 min at 20°C. Wells were then washed 6 times with PBS-Tween 20 and developed with 100 μL of pNPP substrate at 1 mg/mL (Sigma-Aldrich) and read at 405 nm.

Peptide ELISAs in Figure S3E were carried out with a set of sixty-two custom-synthesized biotinylated 20-mer peptides of PfRH5 overlapping by 12 amino acids (Mimotopes). The list of peptide sequences was described previously by Payne et al., 2017. Briefly, the peptides were designed to be oriented and tethered by their N-terminal biotinylated linker peptide (biotin-SGSG) with the exception the first peptide which contained the biotinylated linker at its C terminus to preserve potential binding activity to the most N-terminal residues. These peptides were coated to the bottom wells of a streptavidin-coated 96-well plate in 50 μL. The next day wells were blocked with 200 μL of Blocker Casein (Thermo Fisher Scientific) and washed five times with PBS-Tween 20. Bound mAbs were detected using an anti-human gamma-chain-specific alkaline phosphatase-conjugated secondary antibody (Sigma-Aldrich) or the mouse equivalent for QA1 in 50 μL for 30 min at a dilution of 1:1000 in PBS and washed six times with PBS-Tween 20 before being developed with 100 μL of pNPP alkaline phosphatase substrate for 20 min and read at 405 nm. To determine total PfRH5FL-specific IgG as in Figures 3F and 3I, standardized methodology was used as described previously (Sheehy et al., 2011, Payne et al., 2017). Responses measured in AU are reported in μg/mL following generation of a conversion factor by calibration-free concentration analysis (CFCA). For the calculation of the conversion factor, see Williams et al. (2012).

#### Animal immunization

In Figures 3F–3H and S5B using PfRH5-reactive IgG, two groups of six outbred New Zealand White rabbits were immunized three times three weeks apart and terminally exsanguinated two weeks following the final vaccination. Vaccines were formulated as equimolar doses (29 μg of PfRH5FL or 20 μg of PfRH5ΔNL per dose) in 50% v/v AddaVax adjuvant (InvivoGen), a squalene-based oil-in-water nano-emulsion and administered by intramuscular (i.m.) route by GenScript. In Figures 5E and S5F, anti-PfAMA1, anti-PfMSP1 and anti-PfRH4 antisera were generated in rabbits as previously reported (Douglas et al., 2011, Williams et al., 2012). For PfCyRPA, rabbit immunizations to generate the IgG used in Figure 5E were carried out by Cambridge Research Biochemicals. New Zealand white female rabbits (n = 2) were immunized i.m. on day 0 with 100 μg of PfCyRPA protein formulated in AddaVax adjuvant (InvivoGen) followed by two i.m. booster immunizations on days 28 and 56. Serum was collected 1 week after the final immunization on day 63. To generate the PfCyRPA-reactive IgG used in Figure S5F, a single Sprague Dawley rat was immunized three times on day 0, day 28, day 56 and terminally exsanguinated two weeks following the final vaccination. Vaccines were formulated as 50 μg doses in complete Freund’s adjuvant for the initial vaccination and incomplete Freund’s adjuvant for the following two, and administered i.m by GenScript. To generate the PfRipr-reactive IgG used in Figures 5E and S5F, a single New Zealand White rabbit was immunized four times on day 0, day 14, day 28, day 42 and terminally exsanguinated two weeks following the final vaccination. Vaccines were formulated as 20 μg doses in complete Freund’s adjuvant for the initial vaccination and incomplete Freund’s adjuvant for the following three, and administered i.m by GenScript.

#### Affinity determination by SPR

Data were collected on a Biacore X100 (GE Healthcare). Experiments were performed at 25°C in Dulbecco’s PBS + 0.005% Polysorbate-20 (GE Healthcare). In Figure 1B and Figure S1B, a sensor chip protein A (GE Healthcare) was used to capture 50-100 RU of purified mAb diluted in SPR running buffer at a flow rate of 5 μL/min on flow cell 2. Next, an appropriate range (typically 20 nM-0.625 nM) of six 2-fold dilutions with one replicate of PfRH5FL was injected for 90 s at 60 μL/min and dissociation was measured for 1600 s (7200 s when necessary). The PfRH5FL analyte was > 95% pure as assessed by SDS-PAGE. Specific binding of the PfRH5FL protein to mAb was obtained by reference-subtracting the response of a blank surface from that of the mAb-coated surface. The sensor surface was regenerated with a 60 s pulse of 10 mM glycine-HCl pH 1.5 (GE Healthcare). In Figure S6E, sensor chips CM5 (GE Healthcare) were amine-coupled with approximately 90 RU of R5.004 or R5.016 antibody on flow cell 2. Five two-fold dilutions of PfRH5FL or PfRH5FL-R5.011 Fab fragment complex (made by co-incubating PfRH5FL with a 3-fold molar excess of R5.011 Fab fragment for 30 min at room temperature; all PfRH5FL was bound by R5.011 Fab fragment as assessed by SEC) were injected for 90 s at 60 μL/min at concentrations ranging from 10 nM to 0.625 nM and dissociation was measured for 800 s. The R5.004 sensor chip surface was regenerated with a 30 s pulse of 10 mM glycine-HCl pH 3.0 (GE Healthcare) whereas the R5.016 sensor chip surface was regenerated with a 30 s pulse of 10 mM glycine-HCl pH 1.5. Sensorgrams were fitted to a global Langmuir 1:1 interaction model, allowing determination of the kinetic association and dissociation rate constants using Biacore X100 evaluation software.

#### AVEXIS blocking assay

All AVEXIS assays were conducted at the Wellcome Trust Sanger Institute, Cambridge, UK (Bushell et al., 2008). Biotinylated monomeric bait protein was captured on streptavidin-coated flat-bottomed 96-well microtiter plates in 50 μL volumes for 1 h at 20°C. Plates were then washed 3 times with PBS and incubated with 50 μL of human PfRH5-specific mAb for 1 h at 20°C. The plates were washed 3 more times in PBS before the addition of 50 μL of pentameric β-lactamase-tagged prey proteins for a further 1 h at 20°C. All wells were subsequently washed twice with PBS-Tween 20 and then twice with PBS before the addition of 150 μL/well of the β-lactamase substrate nitrocefin. Absorbance readings were made at 485 nm. The protein constructs were all full-length ectodomains with threonine to alanine mutations to remove N-linked glycosylation sequons (except for basigin) as previously reported (Crosnier et al., 2011, Galaway et al., 2017): PfRH5 amino acids F25-Q526; PfCyRPA amino acids D29–E362; PfP113 amino acids Y23-K942; and basigin isoform 2 amino acids M1-A23 followed by G140-L322. All bait and prey proteins were expressed as fusion proteins N-terminal of a CD4d3+4 tag, a biotin acceptor peptide and a His6 tag. Prey proteins were expressed with a C-terminal with a collagen oligomeric matrix protein (COMP) peptide to promote pentamerization and a β-lactamase enzyme for quantification purposes.

#### Assay of GIA

All mAb GIA assays in Figures 2A–2C and 3E were performed at the GIA Reference Center, NIAID, NIH. Test mAbs were buffer exchanged against RPMI 1640 (KD Medical) and concentrated to 6 mg/mL. The one-cycle GIA was performed at indicated concentrations of mAbs in duplicate wells and a biochemical measurement using a *P. falciparum* lactate dehydrogenase assay was used to quantify parasitemia which has been described previously (Malkin et al., 2005). GIA assays performed with rabbit and rat polyclonal antibody and mAb combinations in Figures 3F, 5, and S5 were performed at the Jenner Institute, University of Oxford using identical protocols and procedures to those at the NIAID but in triplicate wells. Polyclonal IgG resulting from animal immunization were pre-incubated with human group O RhD-positive RBC to eliminate spurious GIA results caused by hemagglutination.

#### Live-cell microscopy

Highly synchronous 3D7 parasite cultures at 4% hematocrit were diluted to 0.16% in warmed RPMI media and 0.2 mL of this was added to each well of a Lab-Tek 8-well Chambered Coverglass (Thermo Fisher Scientific). IgGs were added at concentrations indicated for each figure and the chamber was immediately placed on into a preheated (37°C) incubator stage of a Zeiss AxioObserver Z1 fluorescence microscope supplied with humidified gas (94% N_2_, 1% O_2_, and 5% CO_2_). Late stage schizonts that appeared ready to rupture (Crick et al., 2013) were imaged with a Zeiss LCI Plan-NEOFLUAR 63x/1.3 DIC 1 mm Korr objective at 4 frames per second with an AxioCam MRm camera. The schizonts were imaged for 20 minutes and if they did not rupture, a new schizont in a new well was selected. The image files were cropped, time stamped and then converted to AVI video format in Zen microscopy software (Zeiss). The behavior of invading merozoites was manually viewed in FIJI and the invasion statistics were analyzed and graphed in Prism (Graphpad).

#### Dot blot

Briefly, 1.5 μL of anti-PfRH5 mAb, a human anti-*Zaire Ebolavirus* GP IgG1 mAb (α-EBOV) (Rijal et al., 2019), recombinant PfRH5FL (all at 1 mg/mL) and PBS were spotted onto 0.2 μm nitrocellulose membrane and air-dried for 10 min. Afterward, the membrane was blocked in 3% BSA + 3% skimmed milk in PBS for 1 h and washed in PBS. It was then immersed in *P. falciparum* 3D7 culture supernatant for 1 h and washed again in PBS. Bound PfRH5FL was detected by incubating the membrane in PfRH5FL-immunized rabbit serum diluted 2000-fold in PBS followed by an alkaline phosphatase-conjugated anti-rabbit IgG mAb (clone RG-96, Sigma-Aldrich) also diluted 1:2000, separated by two wash steps in PBS. After a final series of five PBS washes, the dot blot was developed with Sigmafast BCIP/NBT alkaline phosphatase substrate at 1 mg/mL (Sigma-Aldrich).

#### Hydrogen-deuterium exchange mass spectrometry

HDX-MS was performed using a Waters HDX platform composed of a liquid handling robotic setup (LEAP technologies) for sample preparation and a nano-Acquity UPLC coupled to a Synapt G2-Si (Waters) mass spectrometer. Samples were prepared by 11-fold dilutions from 7 μM of apo PfRH5FL or PfRH5FL-mAb complex in deuterated or non-deuterated 20 mM HEPES, 150 mM NaCl pH 7.4 buffer. The pH of the sample was brought down to 2.3 by adding 50% vol/vol 150 mM HCl. Non-deuterated and deuterated samples were loaded by the robot. The apo PfRH5FL protein or PfRH5FL-mAb complex was digested in-line using a pepsin-immobilized column at 20°C. The peptides generated from pepsin digestion were trapped on a micro peptide trap for 2 min for the removal of salts at a flow rate of 200 μL/min and then separated using a C18 column with a linear gradient of 5%–80% acetonitrile (CH_3_CN) and water both supplemented with 0.1% formic acid for 12 min at flow rate of 40 μL/min. The liquid chromatography temperature was set at 0°C to reduce back-exchange. Sequence coverage and deuterium uptake were analyzed by using ProteinLynx Global Server (Waters) and DynamX (Waters) programmes, respectively. Peptide mapping was obtained by using nondeuterated samples in triplicates and only unique peptides present in all three data files were selected for deuterium uptake data analysis. Leucine enkephalin at a continuous flow rate of 5 μL/min was sprayed as a lock mass for mass correction. Apo PfRH5FL protein digests provided a list of 2056 peptides, after applying several selection filters and manual inspection only 127 peptides were selected for analysis. These peptides provided > 93% sequence coverage with many overlapping peptides. The samples were labeled for 20 s, 10 min and 2 h. All HDX-MS experiments were performed in duplicate.

### Quantification and Statistical Analysis

Data were analyzed using GraphPad Prism versions 5.04, 7.0.5 and 8.0.1 for Windows (GraphPad Software Inc.). In Figures 2B, 2C, and 3F, a four-parameter sigmoidal dose-response curve was fitted to the relationship between Log10(antibody concentration) and percentage GIA for each dataset and used to interpolate EC_50_ values. In Figure S2, the nonparametric Spearman’s rank correlation coefficient (ρ) was used to assess a correlation between the variables *K*_on_/*K*_off_/*K*_D_ and GIA EC_30_. In Figures 7A, 7B, and S7B, a nonparametric Kolmogorov-Smirnov test was used to compare the cumulative frequency distribution of two groups. In all statistical tests, reported *P*-values are two-tailed with p > 0.05 not considered significant.

### Data and Code Availability

The crystal structures of unliganded R5.004, R5.011 and R5.016 Fab fragments have been deposited in the Protein Data Bank (PDB) under ID codes 6RCO, 6RCQ and 6RCS, respectively. The crystal structure of R5.004 and R5.016 Fab fragments bound to PfRH5 as well as that of R5.011 and R5.016 Fab fragments bound to PfRH5 can be accessed under PDB ID codes 6RCU and 6RCV, respectively.

## References

[bib1] Afonine, P.V., Grosse-Kunstleve, R.W., Echols, N., Headd, J.J., Moriarty, N.W., Mustyakimov, M., Terwilliger, T.C., Urzhumtsev, A., Zwart, P.H., and Adams, P.D. (2012). Towards automated crystallographic structure refinement with phenix.refine. Acta Crystallogr. D Biol. Crystallogr. 68, 352-367. 10.1107/S0907444912001308 PMC3322595 22505256

[bib2] Balazs, A.B., Chen, J., Hong, C.M., Rao, D.S., Yang, L., and Baltimore, D. (2011). Antibody-based protection against HIV infection by vectored immunoprophylaxis. Nature 481, 81-84. 10.1038/nature10660 PMC3253190 22139420

[bib3] Bates, J.T., Keefer, C.J., Slaughter, J.C., Kulp, D.W., Schief, W.R., and Crowe, J.E., Jr. (2014). Escape from neutralization by the respiratory syncytial virus-specific neutralizing monoclonal antibody palivizumab is driven by changes in on-rate of binding to the fusion protein. Virology 454-455, 139-144. 10.1016/j.virol.2014.02.010 PMC4004766 24725940

[bib4] Baum, J., Chen, L., Healer, J., Lopaticki, S., Boyle, M., Triglia, T., Ehlgen, F., Ralph, S.A., Beeson, J.G., and Cowman, A.F. (2009). Reticulocyte-binding protein homologue 5 - an essential adhesin involved in invasion of human erythrocytes by Plasmodium falciparum. Int. J. Parasitol. 39, 371-380. 10.1016/j.ijpara.2008.10.006 19000690

[bib5] Beernink, P.T., Welsch, J.A., Bar-Lev, M., Koeberling, O., Comanducci, M., and Granoff, D.M. (2008). Fine antigenic specificity and cooperative bactericidal activity of monoclonal antibodies directed at the meningococcal vaccine candidate factor h-binding protein. Infect. Immun. 76, 4232-4240. 10.1128/IAI.00367-08 PMC2519416 18591239

[bib6] Boyle, M.J., Reiling, L., Feng, G., Langer, C., Osier, F.H., Aspeling-Jones, H., Cheng, Y.S., Stubbs, J., Tetteh, K.K., Conway, D.J., et al. (2015). Human antibodies fix complement to inhibit Plasmodium falciparum invasion of erythrocytes and are associated with protection against malaria. Immunity 42, 580-590. 10.1016/j.immuni.2015.02.012 PMC4372259 25786180

[bib7] Bricogne, G., Blanc, E., Brandl, M., Flensburg, C., Keller, P., Paciorek, W., Roversi, P., Sharff, A., Smart, O.S., Vonrhein, C., et al. (2011). BUSTER version 2.10. 0 (Global Phasing Ltd.).

[bib8] Bushell, K.M., Sollner, C., Schuster-Boeckler, B., Bateman, A., and Wright, G.J. (2008). Large-scale screening for novel low-affinity extracellular protein interactions. Genome Res. 18, 622-630. 10.1101/gr.7187808 PMC2279249 18296487

[bib9] Bustamante, L.Y., Bartholdson, S.J., Crosnier, C., Campos, M.G., Wanaguru, M., Nguon, C., Kwiatkowski, D.P., Wright, G.J., and Rayner, J.C. (2013). A full-length recombinant Plasmodium falciparum PfRH5 protein induces inhibitory antibodies that are effective across common PfRH5 genetic variants. Vaccine 31, 373-379. 10.1016/j.vaccine.2012.10.106 PMC3538003 23146673

[bib10] Bustamante, L.Y., Powell, G.T., Lin, Y.C., Macklin, M.D., Cross, N., Kemp, A., Cawkill, P., Sanderson, T., Crosnier, C., Muller-Sienerth, N., et al. (2017). Synergistic malaria vaccine combinations identified by systematic antigen screening. Proc. Natl. Acad. Sci. USA 114, 12045-12050. 10.1073/pnas.1702944114 PMC5692528 29078270

[bib11] Campeotto, I., Goldenzweig, A., Davey, J., Barfod, L., Marshall, J.M., Silk, S.E., Wright, K.E., Draper, S.J., Higgins, M.K., and Fleishman, S.J. (2017). One-step design of a stable variant of the malaria invasion protein RH5 for use as a vaccine immunogen. Proc. Natl. Acad. Sci. USA 114, 998-1002. 10.1073/pnas.1616903114 PMC5293100 28096331

[bib12] Chen, L., Lopaticki, S., Riglar, D.T., Dekiwadia, C., Uboldi, A.D., Tham, W.H., O’Neill, M.T., Richard, D., Baum, J., Ralph, S.A., and Cowman, A.F. (2011). An EGF-like protein forms a complex with PfRh5 and is required for invasion of human erythrocytes by Plasmodium falciparum. PLoS Pathog. 7, e1002199. 10.1371/journal.ppat.1002199 PMC3164636 21909261

[bib13] Chen, L., Xu, Y., Healer, J., Thompson, J.K., Smith, B.J., Lawrence, M.C., and Cowman, A.F. (2014). Crystal structure of PfRh5, an essential P. falciparum ligand for invasion of human erythrocytes. eLife 3, e04187. 10.7554/eLife.04187 PMC4356141 25296023

[bib14] Correia, B.E., Bates, J.T., Loomis, R.J., Baneyx, G., Carrico, C., Jardine, J.G., Rupert, P., Correnti, C., Kalyuzhniy, O., Vittal, V., et al. (2014). Proof of principle for epitope-focused vaccine design. Nature 507, 201-206. 10.1038/nature12966 PMC4260937 24499818

[bib15] Cowman, A.F., Tonkin, C.J., Tham, W.H., and Duraisingh, M.T. (2017). The Molecular Basis of Erythrocyte Invasion by Malaria Parasites. Cell Host Microbe 22, 232-245. 10.1016/j.chom.2017.07.003 PMC12801281 28799908

[bib16] Crick, A.J., Tiffert, T., Shah, S.M., Kotar, J., Lew, V.L., and Cicuta, P. (2013). An automated live imaging platform for studying merozoite egress-invasion in malaria cultures. Biophys. J. 104, 997-1005. 10.1016/j.bpj.2013.01.018 PMC3870799 23473482

[bib17] Crosnier, C., Bustamante, L.Y., Bartholdson, S.J., Bei, A.K., Theron, M., Uchikawa, M., Mboup, S., Ndir, O., Kwiatkowski, D.P., Duraisingh, M.T., et al. (2011). Basigin is a receptor essential for erythrocyte invasion by Plasmodium falciparum. Nature 480, 534-537. 10.1038/nature10606 PMC3245779 22080952

[bib18] Douglas, A.D., Williams, A.R., Illingworth, J.J., Kamuyu, G., Biswas, S., Goodman, A.L., Wyllie, D.H., Crosnier, C., Miura, K., Wright, G.J., et al. (2011). The blood-stage malaria antigen PfRH5 is susceptible to vaccine-inducible cross-strain neutralizing antibody. Nat. Commun. 2, 601. 10.1038/ncomms1615 PMC3504505 22186897

[bib19] Douglas, A.D., Williams, A.R., Knuepfer, E., Illingworth, J.J., Furze, J.M., Crosnier, C., Choudhary, P., Bustamante, L.Y., Zakutansky, S.E., Awuah, D.K., et al. (2014). Neutralization of Plasmodium falciparum merozoites by antibodies against PfRH5. J. Immunol. 192, 245-258. 10.4049/jimmunol.1302045 PMC3872115 24293631

[bib20] Douglas, A.D., Baldeviano, G.C., Lucas, C.M., Lugo-Roman, L.A., Crosnier, C., Bartholdson, S.J., Diouf, A., Miura, K., Lambert, L.E., Ventocilla, J.A., et al. (2015). A PfRH5-based vaccine is efficacious against heterologous strain blood-stage Plasmodium falciparum infection in aotus monkeys. Cell Host Microbe 17, 130-139. 10.1016/j.chom.2014.11.017 PMC4297294 25590760

[bib21] Douglas, A.D., Baldeviano, G.C., Jin, J., Miura, K., Diouf, A., Zenonos, Z.A., Ventocilla, J.A., Silk, S.E., Marshall, J.M., Alanine, D.G.W., et al. (2019). A defined mechanistic correlate of protection against Plasmodium falciparum malaria in non-human primates. Nat. Commun. 10, 1953. 10.1038/s41467-019-09894-4 PMC6486575 31028254

[bib22] Draper, S.J., Sack, B.K., King, C.R., Nielsen, C.M., Rayner, J.C., Higgins, M.K., Long, C.A., and Seder, R.A. (2018). Malaria vaccines: recent advances and new horizons. Cell Host Microbe 24, 43-56. 10.1016/j.chom.2018.06.008 PMC6054918 30001524

[bib23] Emsley, P., Lohkamp, B., Scott, W.G., and Cowtan, K. (2010). Features and development of Coot. Acta Crystallogr. D Biol. Crystallogr. 66, 486-501. 10.1107/S0907444910007493 PMC2852313 20383002

[bib24] Foquet, L., Schafer, C., Minkah, N.K., Alanine, D.G.W., Flannery, E.L., Steel, R.W.J., Sack, B.K., Camargo, N., Fishbaugher, M., Betz, W., et al. (2018). Plasmodium falciparum Liver Stage Infection and Transition to Stable Blood Stage Infection in Liver-Humanized and Blood-Humanized FRGN KO Mice Enables Testing of Blood Stage Inhibitory Antibodies (Reticulocyte-Binding Protein Homolog 5) In Vivo. Front. Immunol. 9, 524. 10.3389/fimmu.2018.00524 PMC5861195 29593746

[bib25] Galaway, F., Drought, L.G., Fala, M., Cross, N., Kemp, A.C., Rayner, J.C., and Wright, G.J. (2017). P113 is a merozoite surface protein that binds the N terminus of Plasmodium falciparum RH5. Nat. Commun. 8, 14333. 10.1038/ncomms14333 PMC5309799 28186186

[bib26] Garçon, N., Heppner, D.G., and Cohen, J. (2003). Development of RTS,S/AS02: a purified subunit-based malaria vaccine candidate formulated with a novel adjuvant. Expert Rev. Vaccines 2, 231-238. 10.1586/14760584.2.2.231 12899574

[bib27] Hjerrild, K.A., Jin, J., Wright, K.E., Brown, R.E., Marshall, J.M., Labbe, G.M., Silk, S.E., Cherry, C.J., Clemmensen, S.B., Jorgensen, T., et al. (2016). Production of full-length soluble Plasmodium falciparum RH5 protein vaccine using a Drosophila melanogaster Schneider 2 stable cell line system. Sci. Rep. 6, 30357. 10.1038/srep30357 PMC4960544 27457156

[bib28] Howell, K.A., Brannan, J.M., Bryan, C., McNeal, A., Davidson, E., Turner, H.L., Vu, H., Shulenin, S., He, S., Kuehne, A., et al. (2017). Cooperativity Enables Non-neutralizing Antibodies to Neutralize Ebolavirus. Cell Rep. 19, 413-424. 10.1016/j.celrep.2017.03.049 PMC6082427 28402862

[bib29] Jin, J., Hjerrild, K.A., Silk, S.E., Brown, R.E., Labbe, G.M., Marshall, J.M., Wright, K.E., Bezemer, S., Clemmensen, S.B., Biswas, S., et al. (2017). Accelerating the clinical development of protein-based vaccines for malaria by efficient purification using a four amino acid C-terminal ‘C-tag’. Int. J. Parasitol. 47, 435-446. 10.1016/j.ijpara.2016.12.001 PMC5482323 28153778

[bib30] Jin, J., Tarrant, R.D., Bolam, E.J., Angell-Manning, P., Soegaard, M., Pattinson, D.J., Dulal, P., Silk, S.E., Marshall, J.M., Dabbs, R.A., et al. (2018). Production, quality control, stability, and potency of cGMP-produced Plasmodium falciparum RH5.1 protein vaccine expressed in Drosophila S2 cells. NPJ Vaccines 3, 32. 10.1038/s41541-018-0071-7 PMC6098134 30131879

[bib31] Kabsch, W. (2010). Integration, scaling, space-group assignment and post-refinement. Acta Crystallogr. D Biol. Crystallogr. 66, 133-144. 10.1107/S0907444909047374 PMC2815666 20124693

[bib32] Mahdi Abdel Hamid, M., Remarque, E.J., van Duivenvoorde, L.M., van der Werff, N., Walraven, V., Faber, B.W., Kocken, C.H., and Thomas, A.W. (2011). Vaccination with Plasmodium knowlesi AMA1 formulated in the novel adjuvant co-vaccine HT™ protects against blood-stage challenge in rhesus macaques. PLoS ONE 6, e20547. 10.1371/journal.pone.0020547 PMC3105089 21655233

[bib33] Malkin, E.M., Diemert, D.J., McArthur, J.H., Perreault, J.R., Miles, A.P., Giersing, B.K., Mullen, G.E., Orcutt, A., Muratova, O., Awkal, M., et al. (2005). Phase 1 clinical trial of apical membrane antigen 1: an asexual blood-stage vaccine for Plasmodium falciparum malaria. Infect. Immun. 73, 3677-3685. 10.1128/IAI.73.6.3677-3685.2005 PMC1111886 15908397

[bib34] Manske, M., Miotto, O., Campino, S., Auburn, S., Almagro-Garcia, J., Maslen, G., O’Brien, J., Djimde, A., Doumbo, O., Zongo, I., et al. (2012). Analysis of Plasmodium falciparum diversity in natural infections by deep sequencing. Nature 487, 375-379. 10.1038/nature11174 PMC3738909 22722859

[bib35] McCoy, A.J., Grosse-Kunstleve, R.W., Adams, P.D., Winn, M.D., Storoni, L.C., and Read, R.J. (2007). Phaser crystallographic software. J. Appl. Cryst. 40, 658-674. 10.1107/S0021889807021206 PMC2483472 19461840

[bib36] McLellan, J.S., Correia, B.E., Chen, M., Yang, Y., Graham, B.S., Schief, W.R., and Kwong, P.D. (2011). Design and characterization of epitope-scaffold immunogens that present the motavizumab epitope from respiratory syncytial virus. J. Mol. Biol. 409, 853-866. 10.1016/j.jmb.2011.04.044 PMC3107930 21549714

[bib37] Miura, K., Zhou, H., Diouf, A., Moretz, S.E., Fay, M.P., Miller, L.H., Martin, L.B., Pierce, M.A., Ellis, R.D., Mullen, G.E., and Long, C.A. (2009). Anti-apical-membrane-antigen-1 antibody is more effective than anti-42-kilodalton-merozoite-surface-protein-1 antibody in inhibiting plasmodium falciparum growth, as determined by the in vitro growth inhibition assay. Clin. Vaccine Immunol. 16, 963-968. 10.1128/CVI.00042-09 PMC2708396 19439523

[bib38] Ord, R.L., Caldeira, J.C., Rodriguez, M., Noe, A., Chackerian, B., Peabody, D.S., Gutierrez, G., and Lobo, C.A. (2014). A malaria vaccine candidate based on an epitope of the Plasmodium falciparum RH5 protein. Malar. J. 13, 326. 10.1186/1475-2875-13-326 PMC4152569 25135070

[bib39] Payne, R.O., Silk, S.E., Elias, S.C., Miura, K., Diouf, A., Galaway, F., de Graaf, H., Brendish, N.J., Poulton, I.D., Griffiths, O.J., et al. (2017). Human vaccination against RH5 induces neutralizing antimalarial antibodies that inhibit RH5 invasion complex interactions. JCI Insight 2, e96381. 10.1172/jci.insight.96381 PMC5752323 29093263

[bib40] Pierce, B.G., Boucher, E.N., Piepenbrink, K.H., Ejemel, M., Rapp, C.A., Thomas, W.D., Jr., Sundberg, E.J., Weng, Z., and Wang, Y. (2017). Structure-Based Design of Hepatitis C Virus Vaccines That Elicit Neutralizing Antibody Responses to a Conserved Epitope. J. Virol. 91, JVI.01032-17. 10.1128/JVI.01032-17 PMC5625506 28794021

[bib41] Quan, J., and Tian, J. (2009). Circular polymerase extension cloning of complex gene libraries and pathways. PLoS ONE 4, e6441. 10.1371/journal.pone.0006441 PMC2713398 19649325

[bib42] Reddy, K.S., Pandey, A.K., Singh, H., Sahar, T., Emmanuel, A., Chitnis, C.E., Chauhan, V.S., and Gaur, D. (2014). Bacterially expressed full-length recombinant Plasmodium falciparum RH5 protein binds erythrocytes and elicits potent strain-transcending parasite-neutralizing antibodies. Infect. Immun. 82, 152-164. 10.1128/IAI.00970-13 PMC3911863 24126527

[bib43] Reddy, K.S., Amlabu, E., Pandey, A.K., Mitra, P., Chauhan, V.S., and Gaur, D. (2015). Multiprotein complex between the GPI-anchored CyRPA with PfRH5 and PfRipr is crucial for Plasmodium falciparum erythrocyte invasion. Proc. Natl. Acad. Sci. USA 112, 1179-1184. 10.1073/pnas.1415466112 PMC4313826 25583518

[bib44] Rijal, P., Elias, S.C., Machado, S.R., Xiao, J., Schimanski, L., O’Dowd, V., Baker, T., Barry, E., Mendelsohn, S.C., Cherry, C.J., et al. (2019). Therapeutic Monoclonal Antibodies for Ebola Virus Infection Derived from Vaccinated Humans. Cell Rep. 27, 172-186. 10.1016/j.celrep.2019.03.020 30943399

[bib45] Saul, A. (1987). Kinetic constraints on the development of a malaria vaccine. Parasite Immunol. 9, 1-9. 10.1111/j.1365-3024.1987.tb00483.x 2436129

[bib46] Sheehy, S.H., Duncan, C.J., Elias, S.C., Collins, K.A., Ewer, K.J., Spencer, A.J., Williams, A.R., Halstead, F.D., Moretz, S.E., Miura, K., et al. (2011). Phase Ia clinical evaluation of the Plasmodium falciparum blood-stage antigen MSP1 in ChAd63 and MVA vaccine vectors. Mol. Ther. 19, 2269-2276. 10.1038/mt.2011.176 PMC3242658 21862998

[bib47] Singh, S., Miura, K., Zhou, H., Muratova, O., Keegan, B., Miles, A., Martin, L.B., Saul, A.J., Miller, L.H., and Long, C.A. (2006). Immunity to recombinant plasmodium falciparum merozoite surface protein 1 (MSP1): protection in Aotus nancymai monkeys strongly correlates with anti-MSP1 antibody titer and in vitro parasite-inhibitory activity. Infect. Immun. 74, 4573-4580. 10.1128/IAI.01679-05 PMC1539572 16861644

[bib48] Takala, S.L., Coulibaly, D., Thera, M.A., Batchelor, A.H., Cummings, M.P., Escalante, A.A., Ouattara, A., Traore, K., Niangaly, A., Djimde, A.A., et al. (2009). Extreme polymorphism in a vaccine antigen and risk of clinical malaria: implications for vaccine development. Sci. Transl. Med. 1, 2ra5. 10.1126/scitranslmed.3000257 PMC2822345 20165550

[bib49] Tiller, T., Meffre, E., Yurasov, S., Tsuiji, M., Nussenzweig, M.C., and Wardemann, H. (2008). Efficient generation of monoclonal antibodies from single human B cells by single cell RT-PCR and expression vector cloning. J. Immunol. Methods 329, 112-124. 10.1016/j.jim.2007.09.017 PMC2243222 17996249

[bib50] Volz, J.C., Yap, A., Sisquella, X., Thompson, J.K., Lim, N.T., Whitehead, L.W., Chen, L., Lampe, M., Tham, W.H., Wilson, D., et al. (2016). Essential Role of the PfRh5/PfRipr/CyRPA Complex during Plasmodium falciparum Invasion of Erythrocytes. Cell Host Microbe 20, 60-71. 10.1016/j.chom.2016.06.004 27374406

[bib51] Walter, T.S., Meier, C., Assenberg, R., Au, K.-F., Ren, J., Verma, A., Nettleship, J.E., Owens, R.J., Stuart, D.I., and Grimes, J.M. (2006). Lysine methylation as a routine rescue strategy for protein crystallization. Structure 14, 1617-1622. 10.1016/j.str.2006.09.005 PMC7126202 17098187

[bib52] Wanaguru, M., Liu, W., Hahn, B.H., Rayner, J.C., and Wright, G.J. (2013). RH5-Basigin interaction plays a major role in the host tropism of Plasmodium falciparum. Proc. Natl. Acad. Sci. USA 110, 20735-20740. 10.1073/pnas.1320771110 PMC3870751 24297912

[bib53] Weiss, G.E., Gilson, P.R., Taechalertpaisarn, T., Tham, W.H., de Jong, N.W., Harvey, K.L., Fowkes, F.J., Barlow, P.N., Rayner, J.C., Wright, G.J., et al. (2015). Revealing the sequence and resulting cellular morphology of receptor-ligand interactions during Plasmodium falciparum invasion of erythrocytes. PLoS Pathog. 11, e1004670. 10.1371/journal.ppat.1004670 PMC4344246 25723550

[bib54] Williams, A.R., Douglas, A.D., Miura, K., Illingworth, J.J., Choudhary, P., Murungi, L.M., Furze, J.M., Diouf, A., Miotto, O., Crosnier, C., et al. (2012). Enhancing blockade of Plasmodium falciparum erythrocyte invasion: assessing combinations of antibodies against PfRH5 and other merozoite antigens. PLoS Pathog. 8, e1002991. 10.1371/journal.ppat.1002991 PMC3493472 23144611

[bib55] World Health Organization. (2018). World Malaria Report 2018. (World Health Organization), https://www.who.int/malaria/publications/world-malaria-report-2018/report/en/.

[bib56] Wrammert, J., Smith, K., Miller, J., Langley, W.A., Kokko, K., Larsen, C., Zheng, N.-Y., Mays, I., Garman, L., Helms, C., et al. (2008). Rapid cloning of high-affinity human monoclonal antibodies against influenza virus. Nature 453, 667-671. 10.1038/nature06890 PMC2515609 18449194

[bib57] Wright, G.J., and Rayner, J.C. (2014). Plasmodium falciparum erythrocyte invasion: combining function with immune evasion. PLoS Pathog. 10, e1003943. 10.1371/journal.ppat.1003943 PMC3961354 24651270

[bib58] Wright, K.E., Hjerrild, K.A., Bartlett, J., Douglas, A.D., Jin, J., Brown, R.E., Illingworth, J.J., Ashfield, R., Clemmensen, S.B., de Jongh, W.A., et al. (2014). Structure of malaria invasion protein RH5 with erythrocyte basigin and blocking antibodies. Nature 515, 427-430. 10.1038/nature13715 PMC4240730 25132548

[bib59] Ye, J., Ma, N., Madden, T.L., and Ostell, J.M. (2013). IgBLAST: an immunoglobulin variable domain sequence analysis tool. Nucleic Acids Res. 41, W34-40. 10.1093/nar/gkt382 PMC3692102 23671333

