# Supplementary material for: Human Antibodies that Slow Erythrocyte Invasion Potentiate Malaria-Neutralizing Antibodies
Source: Cell. 2019 Jun 27;178(1):216–228.e21. doi: 10.1016/j.cell.2019.05.025 (PMC6602525; doi:10.1016/j.cell.2019.05.025)
Supplement: Document S1. Tables S1–S4 [file mmc1.pdf]

Cell, Volume *178*

Supplemental Information

Human Antibodies that Slow Erythrocyte Invasion Potentiate Malaria-Neutralizing Antibodies

Daniel G.W. Alanine, Doris Quinkert, Rasika Kumarasingha, Shahid Mehmood, Francesca R. Donnellan, Nana K. Minkah, Bernadeta Dadonaite, Ababacar Diouf, Francis Galaway, Sarah E. Silk, Abhishek Jamwal, Jennifer M. Marshall, Kazutoyo Miura, Lander Foquet, Sean C. Elias, Geneviève M. Labbé, Alexander D. Douglas, Jing Jin, Ruth O. Payne, Joseph J. Illingworth, David J. Pattinson, David Pulido, Barnabas G. Williams, Willem A. de Jongh, Gavin J. Wright, Stefan H.I. Kappe, Carol V. Robinson, Carole A. Long, Brendan S. Crabb, Paul R. Gilson, Matthew K. Higgins, and Simon J. Draper

Table S1

mAb 2

Relative binding

mAb 1

	R5.001	R5.002	R5.003	R5.004	R5.006	R5.007	R5.008	R5.009	R5.010	R5.011	R5.013	R5.014	R5.015	R5.016	R5.017	R5.018	R5.019	c2AC7	c4BA7	c9AD4	QA1
R5.001	0	0	0.642152	0.954113	0.789725	0.952053	0.951768	0.727439	0.893027	0.906644	0.752796	0.957276	0.495795	0.049707	0.701289	0.86404	0.897009	0.873174	0.871574	0.802856	0.841328
R5.002	0.380536	0.040336	0.384995	0.77155	0.3773	0.783765	0.800643	0.373171	0.660892	0.800341	0.577987	0.743963	0.264908	0.78481	0.776059	0.690899	0.810299	0.836836	0.751746	0.774991	0.919271
R5.003	0.65659	0	0	0.919371	0	0.905293	0.964228	0.047561	0.759203	0.970358	0.884049	0.955108	0	0.929299	0.850829	0.879023	0.838319	0.929462	1	0.998607	0.958984
R5.004	1	1	0.906466	0	0.953883	1	0.897508	0.846951	0.875704	0.955366	0	0.967492	0.84289	0.987959	0.152855	0.983907	0	1	0.837184	0.886799	0.307292
R5.006	0.789711	0	0	0.889544	0.015771	0.889153	0.85008	0.046951	0.757038	0.922317	0.828723	0.918266	0	0.919111	0.827256	0.77636	0.790231	0.873174	0.619559	0.815047	0.853516
R5.007	0.790506	0.586134	0.688796	0.763029	0.822461	0.048795	0.784164	0.7375	0.649632	0.878365	0.673926	0.819505	0.79396	0.817845	0.793002	0.676471	0.809163	0.823299	0	0.71543	0.711406
R5.008	0.790133	0.6543	0.787993	0.68323	0.721044	0.781174	0	0.821291	0.005755	0.948556	1	1	0.861698	0.947138	1	0	1	0.990648	0.800114	0.857143	0.642578
R5.009	0.682933	0	0.049987	0.679942	0.049638	0.778506	0.591884	0.010204	0.584631	0.912154	1	0.988141	0	0.880033	0.936331	0.394057	0.911103	0.950568	0.841234	0.853272	0.981439
R5.010	0.906933	0.756043	0.882693	0.858604	0.829369	0.87312	0	0.828737	0	0.003309	1	0.056812	0.760663	0.978362	0.996817	0	0.984648	0.982298	0.906339	0.931386	0.960557
R5.011	0.784	0.565486	0.847777	0.819876	0.797828	0.821931	0.920158	0.97187	0.004739	0	0.873169	0.038886	0.886957	0.942482	1	0.905039	0.951089	0.935204	0.782981	0.824068	1
R5.013	0.827733	0.778527	0.743029	0.137377	0.76758	0.908782	0.975916	0.952289	0.933311	0.943442	0	0.987865	0.895238	0.963298	0.006765	0.902455	0.027133	0.97328	0.87036	0.910978	0.280742
R5.014	0.771733	0.373806	0.781211	0.788089	0.735264	0.767831	0.922798	0.968009	0	0	0.904981	0	0.773085	0.908244	0.932352	0.861757	0.96894	0.917502	0.843518	0.826531	0.996133
R5.015	0.692566	0	0.025124	0.823627	0.125308	0.962939	0.901929	0.155452	0.848079	0.96091	0.970269	1	0	0.960534	0.981174	1	0.945316	0.955851	0.754032	0.909643	0.79041
R5.016	0	0.70901	0.700165	0.858985	0.810653	0.858727	0.981535	0.831776	0.863617	0.928216	0.967982	0.958922	0.707371	0.014572	0.958749	0.920936	0.881069	0	0.764401	0	0.851508
R5.017	0.98908	0.867061	0.80562	0.014143	0.885776	1	1	0.957944	0.848079	0.958778	0	1	0.858971	1	0	1	0	1	1	0.994268	0.055105
R5.018	1	1	0.852562	0.837354	0.868534	0.951536	0.125154	0.891277	0.116962	0.928927	1	0.975353	0.878999	1	1	0	0.969103	0.974128	1	0.949427	0.665316
R5.019	0.952121	0.998523	0.790744	0	0.815271	0.942984	1	0.936137	0.890375	0.930348	0	0.987841	0.872601	0.956082	0	0.819685	0	0.993591	0.971774	0.938301	0
c2AC7	1	1	0.879339	0.910982	0.897167	0.934431	0.932704	0.917757	0.903755	0.936389	1	0.960237	0.818915	0.032787	1	0.781767	1	0	1	0	0.211507
c4BA7	0.765806	0.782951	0.84058	0.851134	0.84307	0.049623	0.819299	0.778515	0.823554	0.897797	0.816219	0.9366	0.776456	0.865499	0.927862	0.90689	0.807003	0.946168	0	0.843591	0.847391
c9AD4	0.891134	0.797377	0.797391	0.815217	0.804848	0.833935	0.786527	0.820221	0.776945	0.824124	0.875197	0.851761	0.774405	0.035038	0.880383	0.790503	0.812793	0	0.757787	0	0.30389
QA1	1	1	0.916842	0.014589	0.978158	0.986936	0.733288	0.868794	0.867414	0.925293	0	0.951536	0.816986	0.924409	0.141904	0.766117	0.308992	0.839558	1	0.796996	0

Binding profile correlation

	R5.001	R5.002	R5.003	R5.004	R5.006	R5.007	R5.008	R5.009	R5.010	R5.011	R5.013	R5.014	R5.015	R5.016	R5.017	R5.018	R5.019	c2AC7	c4BA7	c9AD4	QA1
R5.001	1	0.558968	0.297855	-0.44693	0.224137	0.039971	-0.23787	0.247393	-0.16862	-0.0773	-0.30684	-0.06576	0.350491	0.518645	-0.29176	-0.19195	-0.35049	0.224128	0.108745	0.229317	-0.52043
R5.002	0.558968	1	0.858781	-0.51743	0.846204	0.012416	-0.16027	0.818759	-0.001	0.020412	-0.41654	0.047851	0.879592	-0.01383	-0.39685	-0.11201	-0.44901	-0.21319	0.094532	-0.22512	-0.6959
R5.003	0.297855	0.858781	1	-0.34187	0.982884	-0.07777	-0.175	0.968397	-0.2044	-0.26427	-0.30756	-0.24172	0.972951	-0.14059	-0.26491	-0.1176	-0.26467	-0.16253	0.023817	-0.20554	-0.46782
R5.004	-0.44693	-0.51743	-0.34187	1	-0.36647	-0.28957	-0.2118	-0.35988	-0.33835	-0.25283	0.937985	-0.27114	-0.39357	-0.39541	0.939035	-0.24302	0.944588	-0.31125	-0.28405	-0.31329	0.779385
R5.006	0.224137	0.846204	0.982884	-0.36647	1	-0.0868	-0.08977	0.960175	-0.08026	-0.16517	-0.35664	-0.14562	0.955706	-0.20755	-0.31815	-0.03373	-0.3085	-0.19512	-0.01021	-0.23485	-0.51068
R5.007	0.039971	0.012416	-0.07777	-0.28957	-0.0868	1	0.029014	-0.01565	0.053623	0.030516	-0.21208	0.068019	-0.10643	-0.05818	-0.28919	0.009732	-0.25069	-0.04844	0.940859	0.011985	-0.30597
R5.008	-0.23787	-0.16027	-0.175	-0.2118	-0.08977	0.029014	1	-0.05694	0.680479	0.190705	-0.38781	0.16202	-0.14673	-0.26505	-0.3836	0.965629	-0.38633	-0.22174	-0.06649	-0.19908	-0.2075
R5.009	0.247393	0.818759	0.968397	-0.35988	0.960175	-0.01565	-0.05694	1	-0.1926	-0.28481	-0.33416	-0.2607	0.978032	-0.14733	-0.31058	-0.01659	-0.3032	-0.16179	0.07772	-0.19802	-0.47818
R5.010	-0.16862	-0.001	-0.2044	-0.33835	-0.08026	0.053623	0.680479	-0.1926	1	0.730097	-0.48296	0.71672	-0.21152	-0.34537	-0.5072	0.66583	-0.50755	-0.29098	-0.07348	-0.25283	-0.48813
R5.011	-0.0773	0.020412	-0.26427	-0.25283	-0.16517	0.030516	0.190705	-0.28481	0.730097	1	-0.28077	0.994465	-0.22163	-0.17025	-0.32121	0.16154	-0.3289	-0.13921	-0.05005	-0.10779	-0.43775
R5.013	-0.30684	-0.41654	-0.30756	0.937985	-0.35664	-0.21208	-0.38781	-0.33416	-0.48296	-0.28077	1	-0.27771	-0.34568	-0.30878	0.98351	-0.40964	0.96776	-0.29846	-0.17605	-0.30859	0.740149
R5.014	-0.06576	0.047851	-0.24172	-0.27114	-0.14562	0.068019	0.16202	-0.2607	0.71672	0.994465	-0.27771	1	-0.19999	-0.17158	-0.32902	0.135476	-0.33754	-0.13025	-0.00915	-0.09955	-0.45597
R5.015	0.350491	0.879592	0.972951	-0.39357	0.955706	-0.10643	-0.14673	0.978032	-0.21152	-0.22163	-0.34568	-0.19999	1	-0.06535	-0.31409	-0.10349	-0.32581	-0.12779	-0.00354	-0.16968	-0.5044
R5.016	0.518645	-0.01383	-0.14059	-0.39541	-0.20755	-0.05818	-0.26505	-0.14733	-0.34537	-0.17025	-0.30878	-0.17158	-0.06535	1	-0.27392	-0.2099	-0.30839	0.866765	-0.02918	0.866756	0.046512
R5.017	-0.29176	-0.39685	-0.26491	0.939035	-0.31815	-0.28919	-0.3836	-0.31058	-0.5072	-0.32121	0.98351	-0.32902	-0.31409	-0.27392	1	-0.38843	0.978506	-0.28617	-0.25936	-0.30688	0.763441
R5.018	-0.19195	-0.11201	-0.1176	-0.24302	-0.03373	0.009732	0.965629	-0.01659	0.66583	0.16154	-0.40964	0.135476	-0.10349	-0.2099	-0.38843	1	-0.40688	-0.18215	-0.1218	-0.16483	-0.21671
R5.019	-0.35049	-0.44901	-0.26467	0.944588	-0.3085	-0.25069	-0.38633	-0.3032	-0.50755	-0.3289	0.96776	-0.33754	-0.32581	-0.30839	0.978506	-0.40688	1	-0.26425	-0.20374	-0.28193	0.753195
c2AC7	0.224128	-0.21319	-0.16253	-0.31125	-0.19512	-0.04844	-0.22174	-0.16179	-0.29098	-0.13921	-0.29846	-0.13025	-0.12779	0.866765	-0.28617	-0.18215	-0.26425	1	-0.03182	0.990411	0.161035
c4BA7	0.108745	0.094532	0.023817	-0.28405	-0.01021	0.940859	-0.06649	0.07772	-0.07348	-0.05005	-0.17605	-0.00915	-0.00354	-0.02918	-0.25936	-0.1218	-0.20374	-0.03182	1	0.036034	-0.30902
c9AD4	0.229317	-0.22512	-0.20554	-0.31329	-0.23485	0.011985	-0.19908	-0.19802	-0.25283	-0.10779	-0.30859	-0.09955	-0.16968	0.866756	-0.30688	-0.16483	-0.28193	0.990411	0.036034	1	0.140655
QA1	-0.52043	-0.6959	-0.46782	0.779385	-0.51068	-0.30597	-0.2075	-0.47818	-0.48813	-0.43775	0.740149	-0.45597	-0.5044	0.046512	0.763441	-0.21671	0.753195	0.161035	-0.30902	0.140655	1

Table S1: Relative binding and binding profile correlation tables. Related to Figure 3.

The “Relative binding” table shows the fraction of mAb2 binding in the presence of bound mAb1. Assays were carried out in both orientations. Boxes are color-coded such that values ≥0.75 are in green, 0.75>X>0.05 are in pink and ≤0.05 are in red. Negative values were normalized to 0 and values >1 were likewise normalized to 1. The “Binding profile correlation” table shows Pearson product-moment correlation values of binding profile (a mAb2 column in the Relative binding table) correlations between each mAb pair. The correlation threshold was set at 0.7; values equal to or above this are colored in orange.

Table S2

	Data collection	R5.004 Fab	R5.011 Fab	R5.016 Fab
Fab fragment structures	**Space group**	P1	$P2_12_12_1$	$P4_32_12$
	**Cell dimensions**			
	a, b, c (Å)	55.21, 70.18, 73.26	47.86, 86.38, 127.40	80.08, 80.08, 162.69
	α, β, γ (°)	101.42, 92.97, 112.47	90, 90, 90	90, 90, 90
	**Wavelength (Å)**	0.9999	0.9786	0.92819
	**Resolution (Å)**	42.45 – 1.66 (1.69-1.66)	47.86 - 2.28 (2.36-2.28)	57.07 – 2.10 (2.14 – 2.10)
	**Total observations**	389497 (18845)	316738 (27746)	397021 (16666)
	**Total unique**	111530 (5493)	24770 (2284)	31807 (1564)
	**R_{pim} (%)**	3.9 (83.9)	7.0 (35.2)	2.9 (55.5)
	**R_{merge} (%)**	6.2 (131.1)	17.1 (83.3)	10.0 (173.1)
	**R_{meas} (%)**	7.3 (156.3)	18.5 (90.6)	10.5 (182.5)
	**$CC_{1/2}$**	0.997 (0.499)	0.995 (0.863)	0.999 (0.536)
	**$I/\sigma(I)$**	8.6 (1.0)	11.0 (3.5)	16.4 (1.3)
	**Completeness (%)**	95.7 (94.5)	99.3 (92.7)	100 (100)
	**Multiplicity**	3.5 (3.4)	12.8 (12.1)	12.5 (10.7)
	**Wilson B factor**	33	45	46
	**Refinement**	**R5.004 Fab**	**R5.011 Fab**	**R5.016 Fab**
	**Reflections**	111520	23457	31616
	**R_{work} / R_{free} (%)**	18.3 / 20.4	18.6 / 22.7	20.4 / 22.3
	**Average B factor**			
	Protein	44.5	39.0	52.5
	Water	51.9	47.0	57.9
	**Number of residues**			
	Protein	872	431	413
	Water	833	238	203
	Ligands	7	2	3
	**R.m.s deviations**			
	Bond lengths (Å)	0.01	0.01	0.01
	Bond angles (°)	1.09	1.23	1.11
	**Ramachandran plot**			
	Favored (%)	97.8	96.7	97.3
	Allowed (%)	2.2	3.3	2.7
	Outliers (%)	0	0	0

	Data collection	PfRH5ΔNL:R5.004:R5.016	PfRH5ΔNL:R5.011:R5.016
Complex structures	**Space group**	C 1 2 1	$P2_12_12_1$
	**Cell dimensions**		
	a, b, c (Å)	235.15, 58.78, 116.79	140.99, 150.99, 163.97
	α, β, γ (°)	90, 106.41, 90	90, 90, 90
	**Wavelength (Å)**	0.9795	0.9763
	**Resolution (Å)**	46.92 – 4.01 (4.07-4.01)	49.16 – 3.58 (3.73-3.58)
	**Total observations**	42257 (1687)	281346 (41777)
	**Total unique**	13131 (522)	41777 (4579)
	**R_{pim} (%)**	8.1 (50.8)	13.6 (51.4)
	**R_{merge} (%)**	12.3 (78.1)	30.3 (114.6)
	**R_{meas} (%)**	14.8 (93.4)	35.8 (135.0)
	**$CC_{1/2}$**	0.988 (0.641)	0.991 (0.863)
	**$I/\sigma(I)$**	5.9 (1.4)	4.7 (1.6)
	**Completeness (%)**	99.0 (80.7)	99.7 (98.4)
	**Multiplicity**	3.2 (3.2)	6.7 (6.7)
	**Wilson B factor**	114	73
	**Refinement**	**PfRH5ΔNL:R5.004:R5.016**	**PfRH5ΔNL:R5.011:R5.016**
	**Reflections**	12992	41468
	**R_{work} / R_{free} (%)**	28.5 / 33.9	28.0 / 30.5
	**Average B factor**		
	PfRH5ΔNL	107.5	65.7
	Fab fragment variable regions	120.8 (R5.004)<br>128.1 (R5.016)	56.6 (R5.011)<br>66.0 (R5.016)
	**Number of residues**		
	Protein	1175	2343
	Water	0	0
	Ligands	0	0
	**R.m.s deviations**		
	Bond lengths (Å)	0.01	0.01
	Bond angles (°)	1.19	1.28
	**Ramachandran plot**		
	Favored (%)	93.0	93.6
	Allowed (%)	7.0	6.4
	Outliers (%)	0	0

Table S2: Crystallographic data collection and refinement statistics of Fab fragments and complexes. Related to Figure 4 and Figure 6.

Table S3

R5.004			PfRH5			Interaction type
FWR/CDR region	Residue (chain)	Group	Structural element	Residue (chain)	Group	
CDR H1	Asn 31 (B)	Side chain	Helix 4	His 365 (A)	Side chain	Hydrogen
CDR H1	Asn 31 (B)	Side chain	Helix 4	Asp 361 (A)	Side chain	Hydrogen
CDR H2	Ile 52 (B)	Side chain	5/6 loop	Trp 447 (A)	Side chain	Hydrophobic
CDR H2	Phe 55 (B)	Side chain	Helix 5	Lys 443 (A)	Side chain	Hydrophobic
CDR H2	Thr 57 (B)	Side chain	Helix 5	Lys 443 (A)	Side chain	Hydrogen
CDR H3	Asp 99 (B)	Side chain	Helix 4	Arg 357 (A)	Side chain	Hydrogen
CDR H3	His 101 (B)	Side chain	Helix 4	Glu 362 (A)	Side chain	Hydrogen
CDR H3	Tyr 105 (B)	Side chain	Helix 4	Asp 361 (A)	Side chain	Hydrogen
CDR L1	Ser 31 (C)	Side chain	1/2 loop	Lys 196 (A)	Side chain	Hydrogen
CDR L1	Ser 31 (C)	Main chain	1/2 loop	Ser 197 (A)	Side chain	Hydrogen
CDR L3	Trp 92 (C)	Side chain	3/4 loop	Asn 352 (A)	Side chain	Hydrogen
CDR L3	Asp 94 (C)	Side chain	3/4 loop	Asn 347 (A)	Side chain	Hydrogen
CDR L3	Asn 97 (C)	Side chain	5/6 loop	Trp 447 (A)	Main chain	Hydrogen
CDR L3	Asn 97 (C)	Side chain	5/6 loop	Arg 448 (A)	Main chain	Hydrogen
**R5.011**			**PfRH5**			**Interaction type**
**FWR/CDR region**	**Residue (chain)**	**Group**	**Structural element**	**Residue (chain)**	**Group**	
CDR H1	Thr 30 (C/H)	Main chain	N-terminal coil	Asn 154 (A/F)	Side chain	Hydrogen
CDR H1	Ser 31 (C/H)	Side chain	N-terminal coil	Ser 153 (A/F)	Main chain	Hydrogen
CDR H1	Ser 31 (C/H)	Main chain	N-terminal coil	Tyr 155 (A/F)	Main chain	Hydrogen
CDR H1	Tyr 32 (C/H)	Side chain	N-terminal coil	Tyr 155 (A/F)	Side chain	Hydrophobic
CDR H2	Asn 52 (C/H)	Side chain	N-terminal coil	Asn 156 (A/F)	Side chain	Hydrogen
CDR H2	Thr 53 (C/H)	Main chain	N-terminal coil	Asn 156 (A/F)	Side chain	Hydrogen
CDR H2	Thr 53 (C/H)	Side chain	N-terminal coil	Asn 156 (A/F)	Side chain	Hydrogen
CDR H2	Asn 54 (C/H)	Main chain	N-terminal coil	Asn 156 (A/F)	Side chain	Hydrogen
CDR H3	Asn 102 (C/H)	Main chain	N-terminal coil	Asn 159 (A/F)	Main chain	Hydrogen
CDR H3	Tyr 104 (C/H)	Main chain	Beta-strand 1	Ile 161 (A/F)	Main chain	Hydrogen
CDR H3	Ser 106 (C/H)	Main chain	Beta-strand 1	Ile 161 (A/F)	Main chain	Hydrogen
CDR H3	Ser 106 (C/H)	Side chain	Beta-strand 1	Ile 163 (A/F)	Main chain	Hydrogen
CDR H3	Ser 107 (C/H)	Side chain	Helix 3	Lys 311 (A/F)	Side chain	Hydrogen
CDR H3	Tyr 109 (C/H)	Side chain	Helix 3	Asn 308 (A/F)	Side chain	Hydrogen
CDR H3	Ser 111 (C/H)	Side chain	Helix 3	Lys 319 (A/F)	Side chain	Hydrogen
CDR H3	Tyr 114 (C/H)	Side chain	Helix 3	Lys 319 (A/F)	Side chain	Hydrogen
CDR L1	Gly 28 (B/G)	Main chain	Helix 3	Lys 312 (A/F)	Side chain	Hydrogen
CDR L1	Gly 28 (B/G)	Main chain	Helix 3	Asn 308 (A/F)	Side chain	Hydrogen
CDR L1	Ser 29 (B/G)	Side chain	Helix 3	Asn 308 (A/F)	Side chain	Hydrogen
CDR L2	Asp 49 (B/G)	Side chain	Helix 3	Lys 316 (A/F)	Side chain	Hydrogen
CDR L2	Asp 50 (B/G)	Side chain	Helix 3	Lys 312 (A/F)	Side chain	Hydrogen
CDR L2	Asp 52 (B/G)	Main chain	Helix 3	Lys 316 (A/F)	Main chain	Hydrogen
Light chain FWR3	Asn 65 (B/G)	Side chain	Helix 3	Lys 312 (A/F)	Side chain	Hydrogen
Light chain FWR3	Gly 67 (B/G)	Main chain	Helix 3	Asp 305 (A/F)	Side chain	Hydrogen
**R5.016**			**PfRH5**			**Interaction type**
**FWR/CDR region**	**Residue (chain)**	**Group**	**Structural element**	**Residue (chain)**	**Group**	
CDR H1	Thr 28 (E)	Side chain	Helix 2	Lys 211 (A)	Side chain	Hydrogen
CDR H1	Ser 31 (E)	Side chain	Helix 2	Asp 207 (A)	Side chain	Hydrogen
CDR H1	Tyr 32 (E)	Side chain	Helix 2	Ala 208 (A)	Main chain	Hydrogen
CDR H2	Tyr 54 (E)	Side chain	Helix 2	Ile 204 (A)	Side chain	Hydrophobic
Heavy chain FWR3	Arg 98 (E)	Side chain	Helix 2	Glu 215 (A)	Side chain	Hydrogen
CDR H3	Pro 101 (E)	Side chain	Helix 2	Ile 204 (A)	Side chain	Hydrophobic
CDR H3	Gln 102 (E)	Side chain	Helix 2	Gly 201 (A)	Main chain	Hydrogen
CDR H3	Asp 105 (E)	Side chain	Helix 2	Lys 202 (A)	Side chain	Hydrogen
CDR H3	Tyr 111 (E)	Side chain	Helix 2	Lys 202 (A)	Side chain	Hydrophobic
CDR H3	Tyr 111 (E)	Main chain	Helix 3	Tyr 335 (A)	Side chain	Hydrogen
CDR H3	Tyr 113 (E)	Side chain	Helix 3	Asp 331 (A)	Side chain	Hydrogen
CDR H3	Tyr 113 (E)	Side chain	Helix 2	Phe 209 (A)	Side chain	Hydrophobic
CDR H3	Asp 117 (E)	Side chain	Helix 2	Lys 212 (A)	Side chain	Hydrogen
CDR L2	Leu 54 (D)	Main Chain	Helix 3	Lys 327 (A)	Side chain	Hydrogen
CDR L2	Glu 55 (D)	Side chain	Helix 2	Lys 212 (A)	Side chain	Hydrogen
CDR L2	Ser 56 (D)	Side chain	Helix 2	Lys 219 (A)	Side chain	Hydrogen

Polymorphic sites within 10 Å of bound mAbs	
**Antibody**	**Substitutions (global frequency)**
R5.004	S197Y (0.164); Y358F (<0.001); H365N (<0.001); I368V (<0.001)
R5.011	D172E (<0.001); V174I (<0.001)
R5.016	C203Y (0.865); I204K (<0.001)

Table S3: Table of proposed interactions between R5.004, R5.011 or R5.016 and PfRH5 and their proximity to known PfRH5 substitutions. Related to Figure 4 and Figure 6.

Substitutions close to the R5.004, R5.011 or R5.016 binding site are defined as non-synonymous mutations catalogued in MalariaGEN (https://www.malariagen.net/apps/pf/4.0/#variation) whose α-carbon is within 10 Å of any mAb atom. Natural variation in PfRH5 sequence is unlikely to greatly affect R5.004, R5.011 or R5.016 binding as all known mutations close to their binding site are either rare or unaffected by the mutation (**Figure 1C**).

Table S4

	Primer number	Primer sequence (5' to 3')
**RT and PCR1**	1	ACAGGTGCCCACTCCCAGGTGCAG
	2	AAGGTGTCCAGTGTGARGTGCAG
	3	CCCAGATGGGTCCTGTCCCAGGTGCAG
	4	CAAGGAGTCTGTTCCGAGGTGCAG
	5	GGAAGGTGTGCACGCCGCTGGTC
	6	ATGAGGSTCCCYGCTCAGCTGCTGG
	7	CTCTTCCTCCTGCTACTCTGGCTCCCAG
	8	ATTTCTCTGTTGCTCTGGATCTCTG
	9	GTTTCTCGTAGTCTGCTTTGCTCA
	10	GGTCCTGGGCCCAGTCTGTGCTG
	11	GGTCCTGGGCCCAGTCTGCCCTG
	12	GCTCTGTGACCTCCTATGAGCTG
	13	GGTCTCTCTCSCAGCYTGTGCTG
	14	GTTCTTGGGCCAATTTTATGCTG
	15	GGTCCAATTCYCAGGCTGTGGTG
	16	GAGTGGATTCTCAGACTGTGGTG
	17	CACCAGTGTGGCCTTGTTGGCTTG
**PCR2**	18	CTTTTTCTAGTAGCAACTGCAACCGGTGTACATTCCGAGGTGCAGCTGGTGCAG
	19	CTTTTTCTAGTAGCAACTGCAACCGGTGTACATTCTGAGGTGCAGCTGGTGGAG
	20	CTTTTTCTAGTAGCAACTGCAACCGGTGTACATTCCCAGGTGCAGCTGCAGGAG
	21	CTTTTTCTAGTAGCAACTGCAACCGGTGTACATTCTGAGGTGCAGCTGTTGGAG
	22	CTTTTTCTAGTAGCAACTGCAACCGGTGTACATTCCCAGGTGCAGCTACAGCAGTG
	23	CTTTTTCTAGTAGCAACTGCAACCGGTGTACATTCCCAGGTTCAGCTGGTGCAG
	24	CTTTTTCTAGTAGCAACTGCAACCGGTGTACATTCCCAGGTCCAGCTGGTACAG
	25	CTTTTTCTAGTAGCAACTGCAACCGGTGTACATTCTGAAGTGCAGCTGGTGGAG
	26	CTTTTTCTAGTAGCAACTGCAACCGGTGTACATTCCCAGGTACAGCTGCAGCAG
	27	CTTTTTCTAGTAGCAACTGCAACCGGTGTACATTCCCAGCTGCAGCTGCAGGAG
	28	CTTTTTCTAGTAGCAACTGCAACCGGTGTACATTCTCAGGTGCAGCTGGTGGAG
	29	GATGGGCCCTTGGTCGACGCTGAGGAGACGGTGACCAG
	30	GATGGGCCCTTGGTCGACGCTGAAGAGACGGTGACCATTG
	31	GATGGGCCCTTGGTCGACGCTGAGGAGACGGTGACCGTG
	32	CTTTTTCTAGTAGCAACTGCAACCGGTGTACATTCTGACATCCAGATGACCCAGTC
	33	CTTTTTCTAGTAGCAACTGCAACCGGTGTACATTCAGACATCCAGTTGACCCAGTCT
	34	CTTTTTCTAGTAGCAACTGCAACCGGTGTACATTGTGCCATCCGGATGACCCAGTC
	35	CTTTTTCTAGTAGCAACTGCAACCGGTGTACATGGGGATATTGTGATGACCCAGAC
	36	CTTTTTCTAGTAGCAACTGCAACCGGTGTACATGGGGATATTGTGATGACTCAGTC
	37	CTTTTTCTAGTAGCAACTGCAACCGGTGTACATTCAGAAATTGTGTTGACACAGTC
	38	CTTTTTCTAGTAGCAACTGCAACCGGTGTACATTCAGAAATAGTGATGACGCAGTC
	39	CTTTTTCTAGTAGCAACTGCAACCGGTGTACATTCAGAAATTGTGTTGACGCAGTCT
	40	CTTTTTCTAGTAGCAACTGCAACCGGTGTACATTCGGACATCGTGATGACCCAGTC
	41	ATGGTGCAGCCACCGTACGTTTGATYTCCACCTTGGTC
	42	ATGGTGCAGCCACCGTACGTTTGATATCCACTTTGGTC
	43	ATGGTGCAGCCACCGTACGTTTAATCTCCAGTCGTGTC
	44	ATGGTGCAGCCACCGTACGTCTGATTTCCACCTTGGTC
	45	CTTTTTCTAGTAGCAACTGCAACCGGTTCCTGGGCCCAGTCTGTGCTGACKCAG
	46	CTTTTTCTAGTAGCAACTGCAACCGGTTCCTGGGCCCAGTCTGCCCTGACTCAG
	47	CTTTTTCTAGTAGCAACTGCAACCGGTTCTGTGACCTCCTATGAGCTGACWCAG
	48	CTTTTTCTAGTAGCAACTGCAACCGGTTCTCTCTCSCAGCYTGTGCTGACTCA
	49	CTTTTTCTAGTAGCAACTGCAACCGGTTCTTGGGCCAATTTTATGCTGACTCAG
	50	CTTTTTCTAGTAGCAACTGCAACCGGTTCCAATTCYCAGRCTGTGGTGACYCAG
	51	GGCTTGAAGCTCCTCACTCGAGGGYGGGAACAGAGTG

Table S4: Table of primers used for the amplification of antibody heavy and light chain variable domain coding sequences. Related to STAR methods.
